# Mechanoresponsive reprogramming of tumor-associated macrophages during cancer progression

**DOI:** 10.3389/fcell.2026.1915582

**Published:** 2026-09-17

**Authors:** Dayeon Kim, Yeonjae Kim, Jihye Lee, Saumendra Kumar Bajpai, Dong-Hwee Kim

**Affiliations:** 1 KU-KIST Graduate School of Converging Science and Technology, Korea University, Seoul, Republic of Korea; 2 Department of Applied Mechanics and Biomedical Engineering, Indian Institute of Technology Madras, Chennai, India; 3 Department of Integrative Energy Engineering, College of Engineering, Korea University, Seoul, Republic of Korea; 4 Biomaterials Research Center, Biomedical Research Institute, Korea Institute of Science and Technology (KIST), Seoul, Republic of Korea

**Keywords:** 3D biomimetic models, cancer-associated fibroblasts, ECM remodeling, macrophage differentiation, TAM-targeted therapy, tumor associated macrophage, tumor microenvironment

## Abstract

Tumor-associated macrophages (TAMs) are critical regulators of the tumor microenvironment. They exhibit diverse pro-tumorigenic and antitumor phenotypes depending on their monocyte-derived or tissue-resident origins. Beyond biochemical signaling, the mechanical properties of the tumor microenvironment, including extracellular matrix stiffness, density, architecture, and spatial heterogeneity, can regulate TAM behavior. This review discusses how these mechanical cues influence TAM infiltration, polarization, and tumor-supportive functions. We further highlight how spatially distinct mechanical environments contribute to TAM heterogeneity within solid tumors. Furthermore, we evaluate how advanced 3D biomimetic cancer platforms, including hydrogels, spheroids, organoids, and microfluidic organ-on-a-chip models, have become essential for accurately recapitulating these mechanoresponsive behaviors and phenotypic adaptations *in vitro*. We also discuss the capacity and current limitations of these models in reproducing the mechanical properties of the tumor microenvironment. Finally, we discuss how understanding these mechanical regulatory pathways may reveal promising avenues for TAM-targeted therapeutic strategies, particularly those aimed at disrupting mechanical niches to achieve effective TAM depletion or antitumor reprogramming. We highlight the need to integrate TAM-targeted strategies with mechanobiological regulation. Collectively, these insights underscore the importance of mechanobiology in regulating macrophage plasticity and its therapeutic potential in clinical oncology.

## 1 Introduction

The tumor microenvironment is a complex and dynamic system composed of cancer cells, immune cells, stromal cells, blood vessel-associated cells, and the extracellular matrix (ECM) (Zhao et al., 2023; Huang et al., 2021). Tumor progression is shaped by interactions between cancer cells and surrounding components, as well as by the mechanical properties of the tumor microenvironment, which can increase tumor invasiveness, metastatic potential, and angiogenesis and thereby enhance therapeutic resistance (Zhang et al., 2021; Xia et al., 2022).

Within the tumor microenvironment, immune cells, particularly tumor-associated macrophages (TAMs), are key regulators because of their diverse functions, which range from antitumor immune responses to tumor-promoting inflammation (Lee Y. S. et al., 2020). TAMs generally arise from two distinct sources: circulating monocytes and tissue-resident macrophages (Casanova-Acebes et al., 2021; Mujal et al., 2022). For monocyte-derived TAMs, circulating monocytes are recruited to the tumor microenvironment by C-C motif chemokine ligand 2 (CCL2), a monocyte-recruiting chemokine secreted by cancer and stromal cells, where they differentiate into macrophages (Yang et al., 2020). Monocytes within the tumor microenvironment tend to acquire an M2-like rather than an M1-like phenotype, thereby exhibiting immunosuppressive and tumor-promoting functions. M2-like TAMs are commonly found in a variety of tumors, including gastric, pancreatic, and head and neck cancers (Heng et al., 2023; Zhang G. et al., 2023; Dong et al., 2025). Tissue-resident TAMs originate from macrophages that are already established within specific tissues, and their TAM phenotype is induced by tumor-derived signals, including the colony-stimulating factor 1 (CSF-1)/colony-stimulating factor 1 receptor (CSF-1R) axis and transforming growth factor-β (TGF-β) signaling (Franzè et al., 2020; Chen et al., 2022).

In addition to cellular interactions, the ECM within the tumor microenvironment can regulate TAM behavior. During cancer progression, the ECM becomes denser and stiffer because of increased deposition of collagen and fibronectin. These changes promote the recruitment and infiltration of monocytes and macrophages through mesenchymal migration and regulate their polarization toward an M2-like TAM phenotype (Wang et al., 2023; Xie et al., 2022a). The resulting accumulation of TAMs facilitates their interactions with cancer-associated fibroblasts (CAFs) within the tumor microenvironment, establishing a positive feedback loop that further promotes ECM stiffening (Maller et al., 2021).

Although conventional two-dimensional (2D) culture systems have been widely used to study TAMs, they have limited capacity to recapitulate cell–cell and cell–matrix interactions and the spatial organization of tumors (Santos et al., 2024). In contrast, 3D culture systems, including hydrogels, organoids, spheroids, and microfluidic chip models, can more effectively recapitulate aspects of TAM behavior within the tumor microenvironment. Compared with 2D culture systems, hydrogel models better mimic cell–ECM interactions, whereas spheroid and organoid models reproduce the three-dimensional (3D) organization of cells. Microfluidic chip models are particularly suitable for monitoring cell infiltration and migration (Ravi et al., 2024; Liu et al., 2024). Collectively, these models provide advanced experimental platforms for investigating monocyte/macrophage recruitment, infiltration, and polarization into TAMs, as well as their contributions to tumor progression.

Recognition of the tumor-promoting roles of TAMs has led to two major therapeutic strategies targeting TAMs: TAM depletion and TAM reprogramming. TAM depletion strategies, such as blocking the CCL2/CCR2 and CSF-1/CSF-1R axes, can inhibit monocyte recruitment and TAM accumulation within the tumor microenvironment, thereby suppressing tumor development (Chen et al., 2022; Ren et al., 2012). In contrast, TAM reprogramming aims to convert M2-like TAMs into M1-like TAMs, thereby transforming the immunosuppressive tumor microenvironment into a more pro-inflammatory state (Jenkins et al., 2018). These strategies have the potential to reduce not only tumor growth but also angiogenesis, metastasis, invasion, and therapeutic resistance.

In this review, we first discuss the origins of TAMs and their functions within the tumor microenvironment, followed by an examination of the biochemical and ECM-derived mechanical cues that regulate TAM behavior. We then explore how 3D culture systems have been used to investigate TAM recruitment, infiltration, and polarization, as well as their tumor-promoting functions. Finally, we review TAM-targeted therapeutic strategies, including TAM depletion and reprogramming, as potential approaches to cancer treatment. We also highlight recent advances in TAM biology from a mechanobiological perspective and discuss the remaining challenges and unresolved questions that must be addressed to better understand interactions between TAMs and the complex tumor microenvironment.

## 2 Origin-specific functional diversity of TAMs

TAMs are not a homogeneous population but rather a heterogeneous group of cells with distinct characteristics and functions that depend on their developmental origins (Franklin et al., 2014). They perform diverse roles within the tumor microenvironment, including promoting tumor growth, immune suppression, angiogenesis, and metastasis (Hirano et al., 2023; Dunsmore et al., 2024). Understanding the diverse functions of TAMs and their relationship to cellular origin provides insight into the heterogeneity of TAM populations and the potential benefits of TAM-targeted therapeutic strategies for cancer patients (Li Y. et al., 2025).

### 2.1 Functions of monocyte-derived TAMs

#### 2.1.1 Developmental origins of monocyte-derived TAMs

Monocyte-derived TAMs arise when circulating monocytes produced in the bone marrow infiltrate tumors. This recruitment process is primarily regulated by two signaling pathways, the CCL2–CCR2 axis and CSF-1/CSF-1R signaling, and involves active recruitment following tumor formation (Yang et al., 2020; Pyonteck et al., 2013). In the CCL2–CCR2 signaling axis, CCL2 secreted by tumor cells binds to CCR2 on the surface of circulating monocytes, directing their chemotaxis toward the tumor (Yang et al., 2020). Notably, CCL2–CCR2-dependent chemotaxis has been validated *in vivo* using a nitrosomethybenzylamine (NMBA)-induced esophageal squamous cell carcinoma (ESCC) carcinogenesis model. Knockout (KO) of either the ligand or receptor demonstrated that both components are essential for monocyte recruitment (Yang et al., 2020). In CCL2-deficient mice, infiltration of CD11b^+^ CCR2^+^ monocytes into tumors was suppressed, resulting in a corresponding reduction in the intratumoral accumulation of CD11b^+^F4/80^+^ TAMs (Yang et al., 2020). The levels of tumor-promoting cytokines typically elevated following NMBA treatment, including interleukin 10 (IL-10), interleukin 12 subunit beta (IL-12β), and IL-13, were also reduced (Yang et al., 2020). In CCR2-deficient mice, infiltration of CD11b^+^ Ly6C^high^ inflammatory monocytes into tumors was completely abolished (Kitamura et al., 2015). At the molecular level, CCL2–CCR2 signaling promotes macrophage extravasation (Kitamura et al., 2015), and the recruited monocytes initially differentiate into metastasis-associated macrophages (MAMs) (Kitamura et al., 2015). Once established within the metastatic niche, CCL2-recruited MAMs secrete CCL3 within the tumor, and CCL3–CCR1 autocrine signaling promotes the perivascular retention of MAMs, thereby facilitating cancer cell intravasation into blood vessels (Kitamura et al., 2015). The second major axis governing monocyte-derived TAM recruitment is CSF-1/CSF-1R signaling, which regulates monocyte recruitment as well as macrophage survival, maturation, and differentiation into TAMs (Kitamura et al., 2015). CSF-1R, a class III receptor tyrosine kinase expressed predominantly on monocytes (MacDonald et al., 2010), is activated by two ligands, CSF-1 and the cytokine IL-34^29^. The effects of anti-CSF-1 treatment on macrophage recruitment have been demonstrated in AE5MG tumor models (MacDonald et al., 2010). Using F4/80 or GFP to identify macrophages, anti-CSF-1 treatment was shown to reduce the number of macrophages within the tumor (MacDonald et al., 2010).

After recruitment, monocytes within tumors retain considerable plasticity and can differentiate into either immunostimulatory antigen-presenting cells or immunosuppressive TAMs, depending on cues from the tumor microenvironment (Devalaraja et al., 2020). These fate decisions are regulated by biochemical and mechanical signals, including cytokines and ECM remodeling. In pancreatic cancer models, lineage-tracing and functional analyses have shown that retinoic acid signaling promotes monocyte differentiation toward TAMs by suppressing interferon regulatory factor 4 (IRF4) (Devalaraja et al., 2020). In these models, IL-13 enhances retinaldehyde dehydrogenase (RALDH) expression, thereby increasing retinoic acid production and facilitating TAM differentiation (Devalaraja et al., 2020). However, this mechanism appears to be context-dependent rather than a universal pathway governing TAM development (Devalaraja et al., 2020).

Consistent with this context-specific differentiation process, single-cell transcriptomic analysis in a pancreatic cancer model identified a continuous differentiation trajectory in which infiltrating monocytes progress through TAM1, TAM2, and TAM3 states rather than following a single, universal pathway of TAM development (Dunsmore et al., 2024). In this pancreatic cancer model, TAM1 cells express CCR2 and CD64, whereas TAM2 cells express CD73, and TAM3 cells express CD36 and CD11c (Dunsmore et al., 2024).

#### 2.1.2 Functions of monocyte-derived TAMs

Monocyte-derived TAMs exhibit greater inflammatory plasticity and migratory capacity, enabling them to respond dynamically to tumor-derived signals (Cui et al., 2018). Consequently, they play important roles in tumor invasion, metastatic dissemination, ECM remodeling, and the establishment of a tumor-supportive microenvironment (Cui et al., 2018; Kim et al., 2019).

Monocyte-derived TAMs promote tumor invasion and metastasis by preferentially localizing to perivascular niches, where they facilitate cancer cell intravasation and vascular remodeling (Arwert et al., 2018). In a mammary tumor model, EdU pulse-chase analysis demonstrated that approximately 40% of newly recruited CD68^+^ TAMs migrated to the perivascular space within 7 days, increasing to approximately 80% by day 10. These findings indicate that monocyte-derived TAMs progressively accumulate around blood vessels during tumor progression (Arwert et al., 2018). This perivascular localization is mediated by CXCR4, which is induced by tumor-derived TGF-β, and CXCL12, which is secreted by perivascular vimentin^+^ fibroblasts (Arwert et al., 2018). Pharmacological inhibition of CXCR4 with AMD3100 prevented TAM accumulation around blood vessels and reduced vascular permeability, highlighting the importance of perivascular localization for TAM function (Arwert et al., 2018). Once localized to the vasculature, monocyte-derived TAMs secrete matrix-degrading enzymes such as matrix metalloproteinase 9 (MMP9), disrupting endothelial tight junctions and facilitating cancer cell extravasation (Kim et al., 2019). Consistent with this mechanism, a 3D microfluidic vascular model incorporating endothelial cells and an ECM scaffold demonstrated that coculture with monocytes reduced the expression of the tight junction proteins ZO-1 and occludin (OCLN), thereby weakening endothelial barrier integrity and increasing vascular permeability (Kim et al., 2019).

Monocyte-derived TAMs also contribute to ECM remodeling and promote tumor progression by inducing epithelial–mesenchymal transition (EMT) in cancer cells (Li et al., 2023). In human colorectal cancer (CRC), a subset of SPP1-expressing monocyte-derived TAMs has been identified as a representative macrophage population associated with ECM remodeling (Li et al., 2023). Using a patient-derived CRC tumoroid–human monocyte coculture model, researchers demonstrated that carcinoma cells polarized monocyte-derived macrophages toward an SPP1^+^ phenotype. These macrophages subsequently induced an EMT transcriptional program through osteopontin–CD44 signaling, thereby promoting tumor progression (Li et al., 2023).

Monocyte-derived TAMs also sustain a tumor-supportive inflammatory microenvironment through reciprocal interactions with cancer cells. In pancreatic ductal adenocarcinoma (PDAC), single-cell RNA sequencing identified monocyte-derived TAMs as the major source of IL-1β within the tumor microenvironment (Caronni et al., 2023). IL-1β released by these cells activates IL1R1-expressing PDAC cells, inducing inflammatory programs that promote tumor growth and macrophage recruitment (Caronni et al., 2023). Accordingly, blockade of IL-1β or genetic deletion of IL1R1 delayed tumor growth, reduced macrophage infiltration, and enhanced CD8^+^ T-cell activation (Caronni et al., 2023). Furthermore, IL-1β stimulation increased the organoid-forming capacity of PDAC cells in an IL1R1-dependent manner, demonstrating that monocyte-derived TAMs establish a positive feedback loop that sustains an inflammatory microenvironment and supports tumor progression (Caronni et al., 2023; Figure 1; Table 1).

**FIGURE 1:**
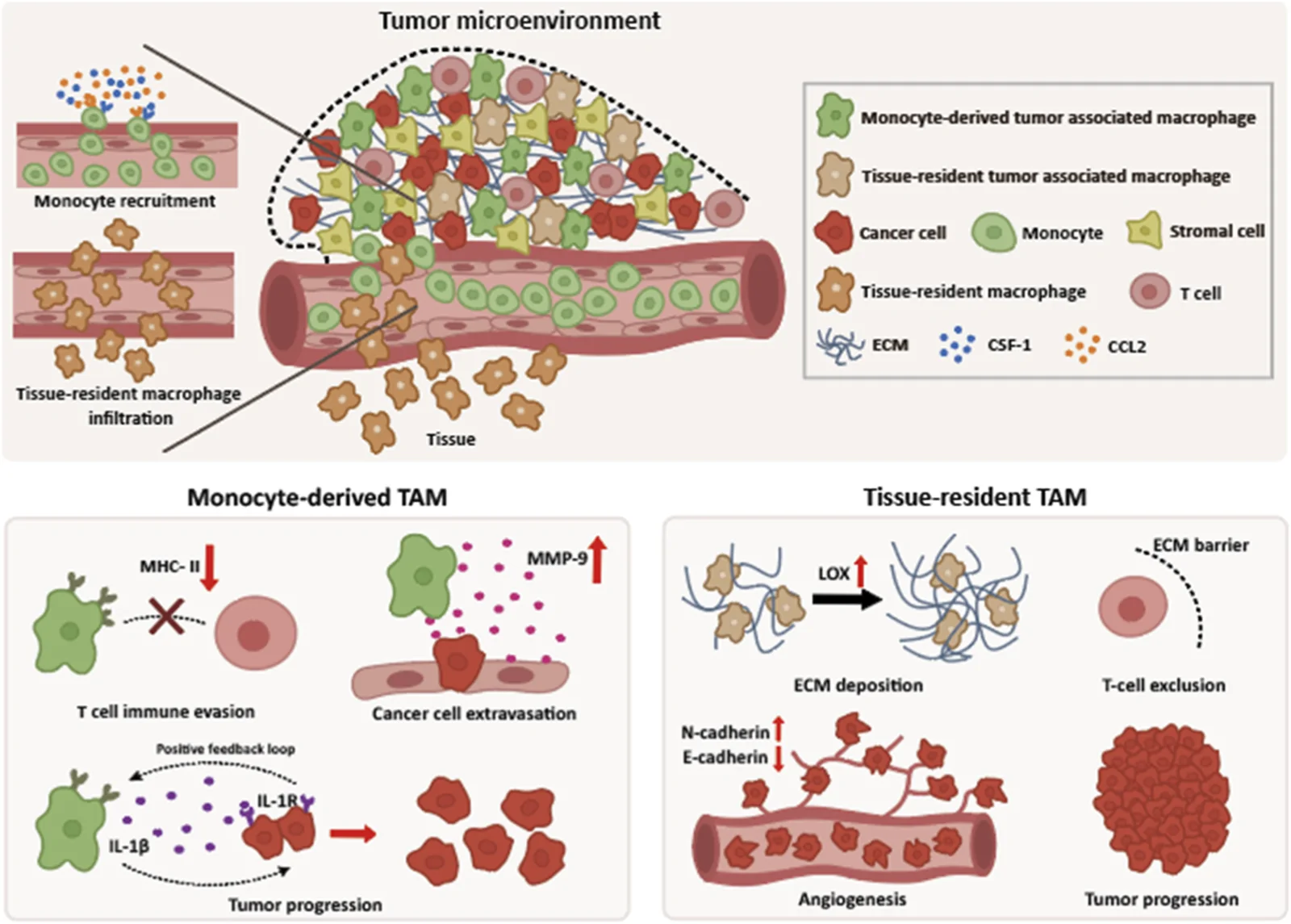
Origin-specific functional diversity of TAMs in the tumor microenvironment. TAMs originate from two major macrophage populations: monocyte-derived and tissue-resident macrophages. Monocyte-derived TAMs arise from circulating monocytes recruited into tumors through signaling pathways such as the CCL2–CCR2 and CSF-1/CSF-1R axes. These macrophages contribute to immune modulation by secreting cytokines and chemokines and suppressing T-cell activity within the tumor microenvironment. In contrast, tissue-resident TAMs arise from macrophages established in specific tissues during development and maintained throughout adulthood through self-renewal. They primarily contribute to fibrosis and ECM remodeling. Through stromal remodeling and matrix deposition, tissue-resident TAMs can indirectly restrict T-cell infiltration and support tumor progression.

**TABLE 1:** Functional heterogeneity of TAMs according to cellular origin.

Category	Monocyte-derived TAMs	Tissue-resident TAMs
Function	Endothelial remodeling Perivascular localization ECM remodeling Inflammatory niche formation	Stromal remodeling and fibrosis Perivascular niche formation Immune regulation
Mechanism	Monocyte-derived macrophages secrete MMP9, reduce ZO-1 and occludin expression, and promote vascular permeability and cancer cell extravasation Tumor-derived TGF-β induces CXCR4 expression in monocyte-derived TAMs, whereas CXCL12 secreted by perivascular fibroblasts recruits TAMs to the perivascular niche Cancer cells polarize monocytes toward an SPP1+ TAM phenotype, which promotes EMT through osteopontin–CD44 signaling IL-1β released by monocyte-derived TAMs activates IL1R1-positive tumor cells, establishing a positive inflammatory feedback loop	Tissue-resident TAMs express collagen, elastin, and LOX, promoting fibrosis and stromal remodeling VEGF induces PLVAP+ endothelial cells, contributing to the establishment of a specialized perivascular niche FOLR2+ macrophages reduce CD8+ T-cell motility and maintain an immunosuppressive stromal niche
Ref.	(Arwert et al., 2018; Li et al., 2023; Caronni et al., 2023)	(Sharma et al., 2020; Zhu et al., 2017; Nalio Ramos et al., 2022)

Although monocyte-derived TAMs have been extensively characterized, several important questions remain unresolved. It remains unclear whether these functional programs are conserved across different tumor types (Dunsmore et al., 2024). Furthermore, how mechanical cues regulate the differentiation trajectory and plasticity of monocyte-derived TAMs following their recruitment into tumors remains poorly understood. In addition, whether mechanical properties of the tumor microenvironment, including ECM stiffness, influence the responsiveness of monocyte-derived TAMs to TAM-targeted therapies remains largely unexplored (Taufalele et al., 2023).

### 2.2 Functions of tissue-resident TAMs

#### 2.2.1 Developmental origins of tissue-resident TAMs

Tissue-resident macrophages are derived from macrophages generated during embryonic development (Hirano et al., 2023). After developing in the yolk sac or fetal liver, these macrophages seed tissues and are maintained into adulthood through self-renewal, forming a relatively independent macrophage population. These macrophages can be reprogrammed by the tumor microenvironment to acquire a TAM phenotype (Casanova-Acebes et al., 2021). In a given organ, the tissue-resident TAMs tend to remain primarily within the tumor stroma and rarely infiltrate densely fibrotic tumor nests (Casanova-Acebes et al., 2021). Within the stromal compartment, they primarily regulate the local microenvironment rather than directly interacting with tumor cells (Zhang et al., 2021). In addition, Notch signaling induces changes in the tumor microenvironment that promote the conversion of monocytes into tissue-resident macrophage-like cells (Sharma et al., 2020). Vascular endothelial growth factor (VEGF) can directly activate plasmalemmal vesicle-associated protein (PLVAP), which regulates endothelial cell fenestration and remodels the tumor microenvironment, thereby promoting the conversion of monocytes into tissue-resident macrophage-like cells (Sharma et al., 2020).

The embryonic origin of tissue-resident TAMs has been directly established through *in vivo* experimental fate-mapping and radiation chimera approaches (Etzerodt et al., 2020). In a mouse metastatic ovarian cancer model generated using ID8-Luc cells, shielded radiation chimeras were used to preserve tissue-resident macrophages (Etzerodt et al., 2020). In this model, CD163^+^ Tim4^+^ omental macrophages were found to be of embryonic origin and maintained independently of bone marrow-derived monocytes throughout tumor development (Etzerodt et al., 2020). Other studies have identified marker expression consistent with an embryonic origin. Across multiple cancer models, tissue-resident TAMs are characterized by the expression of FOLR2, LYVE1, and CD206, markers associated with an embryonic-like macrophage identity, and TAMs expressing these markers are predominantly confined to the tumor stroma (Laviron et al., 2022). In a mammary tumor model, scRNA-seq combined with spatial imaging revealed that stromal macrophages expressing FOLR2 and LYVE1 followed a trajectory distinct from that of monocyte-derived ductal TAMs (Laviron et al., 2022).

#### 2.2.2 Functions of tissue-resident TAMs

Tissue-resident TAMs exhibit a strong capacity for stromal remodeling within the tumor stroma. Consequently, they regulate fibrosis, ECM remodeling, and perivascular niche organization, thereby supporting the establishment of a tumor-promoting stromal environment (Sharma et al., 2020; Zhu et al., 2017). During the early stages of tumor development, tissue-resident TAMs primarily contribute to tissue remodeling, fibrosis, and the establishment of a tumor-supportive stroma (Casanova-Acebes et al., 2021). This phenotype is supported by transcriptional and functional analyses showing that embryonically derived TAMs are enriched in pathways associated with ECM organization and remodeling (Zhu et al., 2017). Consistent with these findings, tissue-resident TAMs highly express ECM components, including collagen and elastin, as well as ECM-remodeling enzymes such as lysyl oxidase (LOX), thereby promoting matrix deposition and stromal remodeling (Zhu et al., 2017). In a PDAC model, tissue-resident TAMs similarly acquire a transcriptional program enriched in fibrosis- and ECM-remodeling-related genes, further supporting their role as key regulators of the tumor stroma rather than direct mediators of antitumor immunity (Zhu et al., 2017).

Tissue-resident TAMs are also preferentially localized within specialized perivascular niches (Sharma et al., 2020). During tumor progression, VEGF signaling induces PLVAP expression in endothelial cells, generating PLVAP^+^ vessels with increased vascular permeability and specialized vascular architecture (Sharma et al., 2020). These fetal-like vascular structures provide supportive perivascular niches in which FOLR2^+^ tissue-resident macrophages preferentially reside and interact with surrounding stromal and immune cells (Nalio Ramos et al., 2022).

Within these niches, FOLR2^+^ tissue-resident macrophages exhibit active membrane remodeling but relatively low motility, enabling them to regulate the spatial organization of immune cells (Nalio Ramos et al., 2022). By reducing CD8^+^ T-cell motility and promoting stable cell–cell interactions, they contribute to an immunosuppressive microenvironment (Ma et al., 2025). These characteristics are closely associated with their embryonic origin and reflect a greater tendency to maintain tissue homeostasis and stromal integrity rather than initiate adaptive immune responses (Zhu et al., 2017) (Figure 1; Table 1).

Despite increasing evidence that tissue-resident TAMs contribute to immune regulation and ECM remodeling, their roles at different stages of tumor progression remain incompletely understood (Casanova-Acebes et al., 2021). In particular, the mechanisms through which tissue-resident TAMs cooperate with newly recruited monocyte-derived TAMs to coordinate stromal remodeling and immune regulation remain unclear (Casanova-Acebes et al., 2021). Furthermore, further research is needed to determine whether tissue-resident TAMs exhibit unique responses to biochemical cues within specialized stromal niches, such as matrix stiffness or vascular remodeling (Wei et al., 2024).

## 3 Mechanical regulation of TAMs

Tumor microenvironment provides diverse mechanical cues, including ECM stiffness, spatial heterogeneity, matrix density, and interstitial fluid pressure, which can influence TAM behavior. These mechanical properties can regulate TAM infiltration, polarization, and functions, thereby promoting tumor progression.

### 3.1 Mechanosensitive TAM infiltration within tumor ECM

The dense and stiff ECM of solid tumors generally acts as a physical barrier that restricts immune cell infiltration. ECM remodeling and collagen reorganization are particularly important because they reshape the physical architecture of tumors, altering the mechanical pathways and niches through which TAMs infiltrate, accumulate, and polarize in response to the tumor microenvironment (Xun et al., 2023; Wu et al., 2024; Marigo et al., 2020a; Yang C. et al., 2024; Bareham et al., 2024). Unlike many other immune cells, macrophages have the ability to migrate through mechanically confined and fibrotic tumor regions. For example, in lymph node metastases of breast cancer, collagen I deposition and the resulting increase in matrix-associated mechanical stress suppressed lymphocyte infiltration, whereas CD68^+^ macrophages were still able to migrate into metastatic regions (Jones et al., 2021). In addition, substrate stiffness regulated YAP-dependent disabled homolog 2 (DAB2) expression in TAMs, thereby promoting haptotaxis, matrix remodeling, and the accumulation of DAB2^+^ TAMs at the invasive front (Marigo et al., 2020b). These findings suggest that TAMs may overcome the restriction of immune cell infiltration imposed by dense ECM because macrophages can sense the mechanical properties of the surrounding ECM and adjust their migration mode accordingly.

Leukocytes generally employ adhesion-independent amoeboid migration, whereas macrophages can undergo adhesion- and proteolysis-dependent mesenchymal migration in response to ECM density and stiffness (Aoun et al., 2020). In inflammatory environments, macrophages primarily exhibit rounded, amoeboid morphology, whereas macrophages in fibrotic tumor microenvironments transition toward integrin-mediated adhesion and ECM degradation-dependent mesenchymal migration (Aoun et al., 2020; Gui et al., 2018). During this transition, macrophages become elongated and form protrusions, thereby strengthening their interactions with the surrounding matrix (Figure 2A; Gui et al., 2018; Barros-Becker et al., 2017). Macrophages have been shown to exhibit ROCK-dependent amoeboid migration outside the tumor boundary but switch to MMP-dependent mesenchymal migration within the dense ECM of the tumor boundary (Gui et al., 2018). This mechanically induced transition in migration mode has been observed in tumor tissues with highly remodeled ECM. In fibrosarcomas, which are surrounded by dense ECM enriched in laminin, fibronectin, and collagen, TAMs exhibited increased migration accompanied by prominent elongation and protrusion formation (Gui et al., 2018). Similarly, as collagen I accumulation increased, macrophages became more elongated, and their protrusions became longer and more stable (Meizlish et al., 2024). Macrophages were also able to infiltrate the dense meshwork formed by collagen IV crosslinking by extending protrusions toward pores, and this migration was significantly suppressed when Rho kinase and myosin II activity were inhibited (Bahr et al., 2022). Furthermore, macrophage infiltration increased as the collagen fiber network became stronger or the thickness of fiber bundles increased (Wang et al., 2023). Collectively, these findings suggest that macrophages actively employ mesenchymal rather than amoeboid migration to adapt to the dense and stiff ECM of the tumor microenvironment.

**FIGURE 2:**
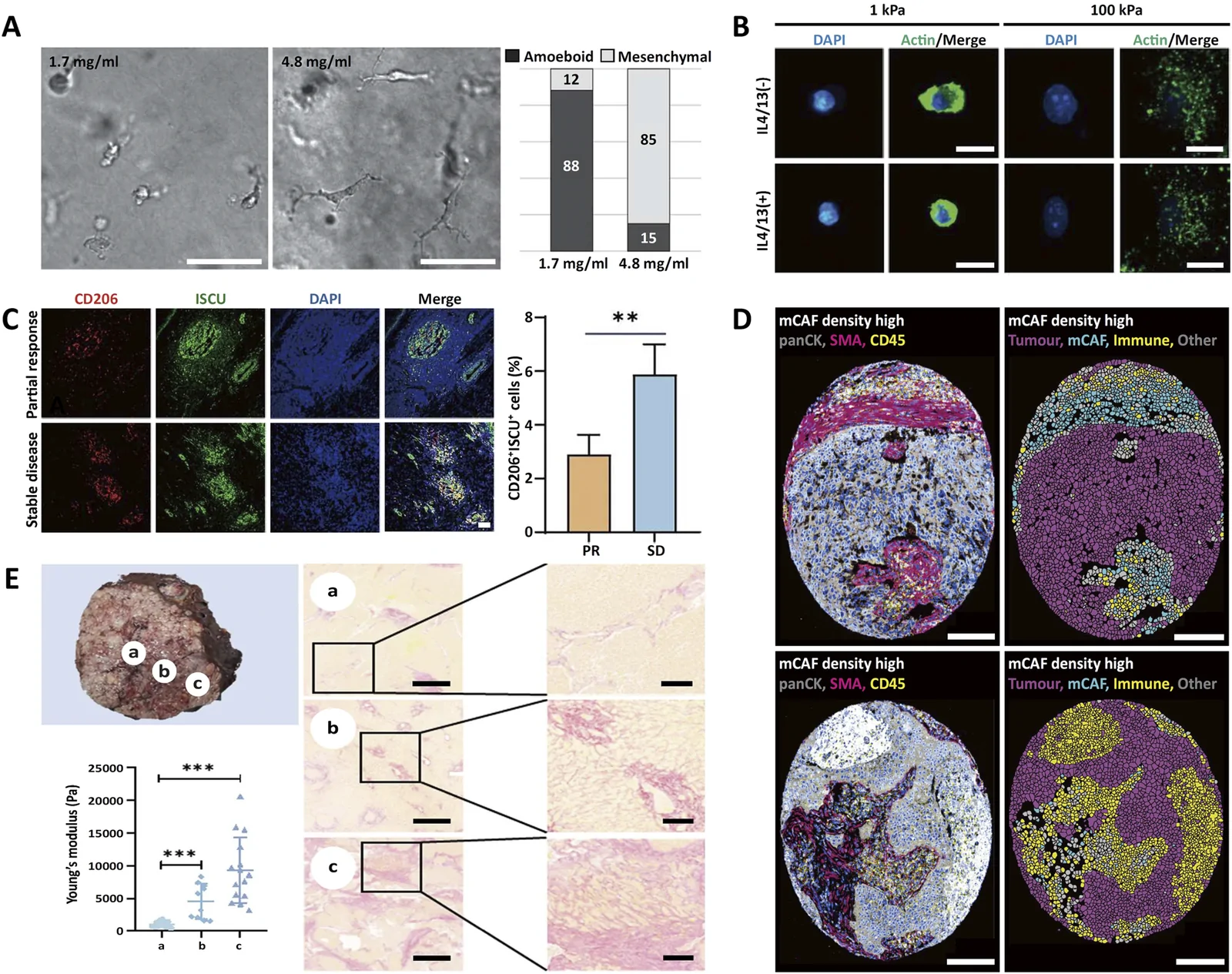
Mechanical regulation of TAM infiltration, polarization, function, and spatial heterogeneity in the tumor microenvironment. **(A)** A dense ECM promotes mesenchymal migration in M2-like macrophages. M2-like macrophages preferentially exhibit an amoeboid morphology in low-density collagen, whereas dense collagen induces a shift toward a mesenchymal morphology (scale bar = 100 µm). Adapted from Čermák et al., *Scientific Data* (2018). **(B)** Increased substrate stiffness promotes the formation of podosome-like actin structures in macrophages. Phalloidin (green) and DAPI (blue) staining illustrates the increased formation of podosome-like actin structures in macrophages cultured on a stiff 100-kPa matrix compared with those cultured on a soft 1-kPa matrix, with or without IL-4/IL-13 treatment (scale bar = 10 µm). This comparison demonstrates stiffness-dependent cytoskeletal mechanosensing in BMDMs regardless of cytokine treatment. Adapted from Shin et al., *Advanced Science* (2025). **(C)** Immunofluorescence staining of the M2 marker CD206 (red) and ISCU (green), with nuclei stained with DAPI (blue), and the proportion of CD206+ISCU + macrophages in esophageal squamous cell carcinoma tumor samples exhibiting partial response (PR) or stable disease (SD) following PD-1 inhibition (scale bar = 100 μm). These findings support an ISCU-mediated mechanism underlying M2-like TAM polarization. Adapted from Luo et al., *Cell Death & Disease* (2025). **(D)** Representative images of non-small cell lung cancer tissues with high or low myofibroblastic CAF (mCAF) density, showing the spatial organization of tumor tissues (magenta), immune cells (yellow), and mCAFs (cyan) (scale bar = 150 μm). Adapted from Cords et al., *Cancer Cell* (2024). **(E)** Stiffness measurements at three sites (groups A, B, and C) on the cross-section of hepatocellular carcinoma tissue. Sirius Red staining shows increased collagen deposition, with Young’s modulus calculated according to the distance from the tumor core (scale bars = 500 μm, left, and 100 μm, right). Adapted from Wei et al., *Journal of Translational Medicine* (2022).

During mesenchymal migration, actin-based protrusive structures known as podosomes play a central role in macrophage movement (Figure 2B). Podosomes are ring-shaped structures composed of an actin core containing microtubules (Herron et al., 2022). Proteins involved in adhesion to ECM components, such as vinculin and integrins, accumulate around the actin core, and podosomes are interconnected through a myosin IIa network (van den Dries et al., 2019). Although podosome cluster size did not significantly change with increasing substrate stiffness, the distance between individual podosomes within clusters decreased, indicating increased podosome density (van den Dries et al., 2019). Additionally, the protrusive force generated by podosomes, which depends on F-actin polymerization and myosin contractility, increased proportionally with substrate stiffness (Labernadie et al., 2014). Podosomes undergo vertical oscillations accompanied by directional flow of actin and vinculin, and podosome clusters formed on stiff substrates exhibited greater coordination of movement than those formed on soft substrates, enabling more efficient collective protrusion (van den Dries et al., 2019). These findings indicate that macrophages can sense substrate stiffness through podosome activity and enhance protrusive activity accordingly. Indeed, macrophages cultured in gelled collagen with higher packing density and stiffness formed membrane protrusions enriched in F-actin and podosome markers, including cortactin, paxillin, gelsolin, and phosphotyrosine proteins, while β1 integrin colocalized with F-actin within these protrusive structures (Van Goethem et al., 2010). Furthermore, collagen degradation activity, detected using the COL2 3/4 C short antibody, was observed at protrusion sites, suggesting that macrophage mesenchymal migration depends on both adhesion and proteolytic activity (Van Goethem et al., 2010).

Mechanistically, macrophage spreading, adhesion, and podosome formation are regulated by the WAVE regulatory complex and Wiskott–Aldrich syndrome protein (WASP)-mediated signaling, which are involved in actin cytoskeletal remodeling and protrusion formation (Stahnke et al., 2021; Leithner et al., 2016). WASP-mediated signaling promotes FAK and paxillin phosphorylation, thereby regulating integrin αM activity and ultimately inducing podosome assembly. Consistent with this mechanism, podosome formation was significantly suppressed in WASP KO macrophages (Stahnke et al., 2021; Leithner et al., 2016). In contrast, protrusion formation was minimal on fibrillar collagen with lower stiffness (Van Goethem et al., 2010). Taken together, these findings indicate that TAMs do not simply passively penetrate the dense and stiff ECM of the tumor microenvironment. Rather, they actively infiltrate the tumor by sensing ECM stiffness and switching from amoeboid to podosome-based mesenchymal migration. Podosome-mediated protrusion formation and ECM degradation may therefore represent key mechanisms underlying this mechanosensitive mode of infiltration (Van Goethem et al., 2010). However, it remains unclear how podosome-mediated mechanosensing is coordinated with other physical cues, including confinement, pore size, and collagen architecture, to determine TAM migration mode and infiltration efficiency.

### 3.2 Stiff tumor microenvironment regulates TAM polarization and functions

Mechanical cues within the tumor microenvironment regulate not only TAM infiltration but also macrophage polarization and function. As a major structural and signaling component of the tumor microenvironment, the ECM can directly induce TAM-associated phenotypes. For example, macrophages cultured in decellularized ECM derived from ovarian cancer metastases acquired a TAM-like phenotype characterized by tissue-remodeling and immunoregulatory features, along with increased expression of ARG1, CD36, macrophage scavenger receptor 1, and macrophage receptor with collagenous structure (Puttock et al., 2023). Among the various properties of the ECM, stiffness is frequently associated with the accumulation of M2-like TAMs that support immune suppression, tissue remodeling, angiogenesis, and tumor progression. In pancreatic ductal adenocarcinoma, increased matrix stiffness decreased the abundance of pro-inflammatory CD8^+^ and CD4^+^ T cells but increased the population of F4/80^+^ macrophages, particularly CD206^+^ M2 macrophages (Zhang H. et al., 2023). Intratumoral heterogeneity in matrix stiffness may induce a phenotypic transition toward an immunoregulatory and wound-healing-associated M2-like phenotype (Laviron et al., 2022; Adlerz et al., 2016; Scott et al., 2021; Meli et al., 2020; Chen et al., 2020; Xie et al., 2022b; Friedemann et al., 2017). Consistent with this, unstimulated macrophages cultured in stiff-matrix *in vitro* systems exhibited increased expression of M2 polarization markers, such as CD206 and IL-10, and reduced expression of M1-associated pro-inflammatory markers, such as IL-12 and TNF-α, a pattern similar to that observed in in vivo hydrogel models (Adlerz et al., 2016; Scott et al., 2021; Meli et al., 2020; Chen et al., 2020; Xie et al., 2022b; Friedemann et al., 2017). In addition, the abundance of α-SMA^+^ CAFs at the invasive front of esophageal squamous cell carcinoma was positively correlated with the abundance of CD163^+^ M2-like macrophages, and higher CAF abundance was associated with the formation of a barrier-like structure that reduced CD4^+^ and CD8^+^ T cell infiltration (Figure 2C; He et al., 2023). Moreover, impaired ECM-mediated mechanical communication between cancer cells and macrophages contributed to tumor immune escape (Yang C. et al., 2024).

A stiff tumor microenvironment may induce M2 polarization of TAMs through coordinated cytoskeletal mechanotransduction. Vimentin-dependent organization of actin stress fibers and podosomes enhances integrin CD11b-mediated podosome turnover, MT1-MMP localization, and ECM degradation capacity, thereby promoting a tissue-remodeling and invasion-supportive M2-like TAM phenotype (Huang et al., 2025). Mechanosensitive TAM polarization in a stiff-matrix microenvironment is also regulated by intracellular signaling mediators, including calcium/calmodulin-dependent kinase kinase 2 (CaMKK2). In breast cancer, CaMKK2 promoted tumor progression by increasing the population of monocytes with impaired antigen-presenting capacity, reducing granzyme B infiltration, decreasing the numbers of CD69-expressing CD8^+^ T cells, B cells, and MHCII + M1-like TAMs, and upregulating CCL17 and CCL22 expression. Collectively, these changes contribute to an anti-inflammatory and pro-tumorigenic tumor microenvironment (Racioppi et al., 2019; Guan et al., 2025).

Clinically, elevated CaMKK2 levels are associated with progressive cancers, including triple-negative breast cancer, and directly correlate with poor patient prognosis (Racioppi et al., 2019; Guan et al., 2025). Culturing bone marrow-derived macrophages (BMDMs) in medium conditioned by CaMKK2-secreting E0771 breast cancer cells downregulated Nos2 and MHCII expression while upregulating Arg1, Chil3/Ym1, and Retnla/Fizz1 expression compared with BMDMs cultured in medium conditioned by CaMKK2-knockdown cells. These changes induced regenerative M2 polarization and anti-inflammatory responses while suppressing T-cell recruitment and proliferation (Racioppi et al., 2019; Guan et al., 2025). Deletion of CaMKK2 did not affect IL-4-induced macrophage polarization but suppressed stiffness-induced M2 polarization, suggesting that CaMKK2 regulates TAM M2 polarization in a stiffness-dependent manner (Guan et al., 2025). Mechanistically, this mechanosensitive activation of CaMKK2 is regulated by phosphorylation of AKT, AMPK, and STAT6 (Guan et al., 2025).

In addition to kinase-mediated signaling, mechanical forces can regulate macrophage polarization through mechanosensitive ion channels. Mechanical tension increased the proportion of CD206^+^ macrophages and enhanced hallmarks of M2 polarization, including decreased inducible nitric oxide synthase (iNOS) levels, increased Arg-1 and IL-10 levels, and upregulation of TGF-β1 mRNA and protein expression (Cai et al., 2023). This response was mediated through Piezo1-dependent calcium signaling (Solis et al., 2019). Piezo1 is a mechanically activated ion channel that converts mechanical stimuli into intracellular Ca^2+^ signals (Solis et al., 2019). The relationship between macrophage polarization and Piezo1 expression may be regulated by p53 activity. Piezo1 knockdown reduced p53 acetylation (Cai et al., 2023), whereas inhibition of the p53 signaling pathway upregulated M2 polarization markers, such as Arg1, IL-10, and VEGFA (Li X. et al., 2025). For example, interaction of cytoplasmic p53 with ISCU relieved p53-dependent suppression of Arg1 transcription, whereas ISCU knockdown retained p53 in the nucleus and reduced CD206 expression. Similarly, MCPIP-1-mediated degradation of p53 enhanced Arg1 expression and M2-like polarization (Luo et al., 2025). However, Piezo1-dependent Ca^2+^ signaling has also been shown to activate inflammatory macrophages (Atcha et al., 2021). Thus, although the direction of the polarization response may depend on the surrounding environment, Piezo1-dependent mechanosensing appears to contribute to macrophage polarization through intracellular Ca^2+^ signaling and interactions with downstream regulators such as p53 (Figure 2D).

Because collagen accumulation and matrix density are major determinants of tumor stiffness, increased collagen deposition and matrix density can reinforce stiffness-associated immunosuppressive M2 polarization. In pancreatic ductal adenocarcinoma, accumulation of collagen and hyaluronic acid increased the abundance of M2-like TAMs, as indicated by decreased iNOS levels and increased TGF-β1, ARG-1, and CD206 levels (Wu et al., 2024). Mimicking the dense ECM of tumors in a 3D culture system activated signaling pathways involved in macrophage M2 polarization, including PI3-AKT, MAPK, TNF, and NF-κB signaling (Wu et al., 2024). In patients, the M1/M2 macrophage ratio was negatively correlated with COL1A1 expression and the matrix index, supporting an association between dense collagen matrices and the M2-like TAM phenotype (Li et al., 2025c; Table 2).

**TABLE 2:** M2 polarization and function of TAMs in various cancer types.

Tumor type	Characterization	Upregulated pathway	Function	Ref.
Breast cancer	CD206^+^	Increased YAP expression	-	(Taufalele et al., 2023)
	Increased Arg1	Ornithine synthesis pathway	Collagen crosslinking	(Tharp et al., 2024)
	Increased CD206, Arg-1	CHI3L1 expression	Inducing cancer metastasis	(Chen et al., 2017)
	CD206^+^/MHC-II^low^	CaMKK2 expression	Inhibiting T cell proliferation	(Racioppi et al., 2019; Guan et al., 2025)
Ovarian cancer	M0, shared signature with M2-like macrophages	Integrin–ECM interactions	Tissue remodeling	(Puttock et al., 2023)
	CD206^+^	-	Suppressing CD8+ T cell infiltration	(Li et al., 2025c)
	Increased CD206, Arg1, MMP19	Oxidative phosphorylation	Inhibiting antigen-specific T-cell function	(Rodriguez-Garcia et al., 2021)
Pancreatic ductal adenocarcinoma	Increased TGF-β1, Arg1, CD206	PI3K–AKT signaling pathway	Immunosuppression	(Wu et al., 2024)
	CD206^+^	Osm expression	Inducing cancer metastasis	(Alonso-Nocelo et al., 2023)
Brain cancer	CD206^+^	Integrin α2β1/NF-κB pathway	Suppressing CD8+ T-cell infiltration	(Wang et al., 2024b)
Colorectal cancer	SPP1^+^	SPP1 signaling pathway	Interaction with CAF	(Xun et al., 2023)
Hepatocellular carcinoma	SPP1^+^	Hypoxia associated pathway	Interaction with CAF	(Liu et al., 2023)
Gastric carcinoma	DAB2^+^	YAP–TAZ activation	ECM remodeling	(Marigo et al., 2020a)
Esophageal squamous cell carcinoma	CD206^+^	Suppressing p53 nuclear translocation	Anti-inflammatory response	(Luo et al., 2025)

Although most current studies have focused on how mechanical cues regulate M1/M2 macrophage polarization, the broader range of phenotypic and functional states of TAMs remains poorly understood. Further studies are needed to determine whether other mechanical cues, including matrix density, collagen accumulation, and spatial stiffness gradients, contribute to TAM heterogeneity and regulate distinct TAM phenotypes and functions.

### 3.3 Spatially heterogeneous mechanical niches shape TAM states

Tumor tissues exhibit substantial spatial heterogeneity in their mechanical properties (Figure 2E). In late-stage MMTV-PyMT tumors, an invasive ductal carcinoma model, matrix stiffness generally increased from the tumor core toward the invasive boundary (Plodinec et al., 2012). Similarly, spatial measurements of hepatocellular carcinoma tissues showed that stiffness progressively increased from the tumor core toward the tumor boundary (Wei et al., 2022).

These differences in stiffness are associated with ECM remodeling at the tumor boundary. In MMTV-PyMT carcinoma tumors, the increase in stiffness from the core to the boundary was accompanied by increased expression of collagen I and lamin-1 proteins (Plodinec et al., 2012). Furthermore, ECM-related pathways, including those involved in ECM organization and collagen fiber organization, were activated near the tumor boundary in colorectal cancer and hepatocellular carcinoma (Xun et al., 2023). Spatial transcriptomic analysis of colorectal cancer further revealed increased expression of genes associated with collagen synthesis and matrix remodeling, including *SPP1*, *MMP11*, and *COL1A1*, in the tumor boundary region (Xun et al., 2023).

Because collagen remodeling and matrix alignment are particularly pronounced at the tumor boundary, region-specific ECM organization may be maintained by spatially localized stromal and immune cell populations. This possibility is supported by spatial transcriptomic analyses showing that tumor boundary remodeling is accompanied by enrichment of specific stromal and myeloid populations associated with ECM organization (Xun et al., 2023). Across multiple cancer types, including colorectal cancer, hepatocellular carcinoma, breast cancer, ovarian cancer, and clear cell renal cell carcinoma, SPP1^+^ macrophages and ASPN^+^ fibroblasts were enriched near the tumor boundary, whereas FOLR2^+^ macrophages and SFRP2^+^ fibroblasts were more abundant in nonmalignant regions (Xun et al., 2023). Functional enrichment analysis showed that ASPN^+^ fibroblasts were associated with ECM assembly, collagen fibril organization, and collagen biosynthetic processes, whereas SPP1^+^ macrophages were associated with angiogenic, migratory, and metabolically active programs (Xun et al., 2023). Moreover, cell–cell interaction analysis revealed strong signaling interactions among SPP1^+^ macrophages, ASPN^+^ fibroblasts, endothelial cells, and tumor cells near the tumor boundary (Xun et al., 2023). These observations suggest that ECM remodeling at the tumor boundary may be supported by coordinated stromal and myeloid programs that reinforce region-specific matrix organization. Notably, ECM-myCAFs spatially colocalized with SPP1^+^ and TREM2^+^ TAM populations and angiogenic endothelial programs, whereas Detox-iCAFs were associated with FOLR2^+^ macrophages in peritumoral regions. These findings suggest that region-specific interactions between CAFs and TAMs with stromal remodeling-associated functions may contribute to the formation of a dense and stiff tumor boundary microenvironment (Croizer et al., 2024).

Collectively, these studies demonstrate that tumors exhibit region-specific mechanical heterogeneity, with tumor boundaries generally displaying greater stiffness than tumor cores (Plodinec et al., 2012; Wei et al., 2022). These spatial differences are closely associated with enhanced ECM remodeling and collagen organization (Xun et al., 2023; Arora et al., 2023). Notably, mechanical heterogeneity within tumors may be actively maintained through interactions between CAFs and TAMs that reinforce localized matrix remodeling and potentially contribute to tumor progression and adverse clinical outcomes (Xun et al., 2023; Croizer et al., 2024). Although spatial mechanical heterogeneity is associated with distinct TAM states, it remains unclear whether mechanical cues or biochemical signals are the primary modulators of TAM localization, phenotypic changes, and ECM remodeling.

## 4 3D models for studying TAMs in mechanically complex tumor microenvironments

In cancer research, the utility of 2D culture systems is limited because these models cannot fully recapitulate the complexity of the tumor microenvironment or adequately represent its cellular heterogeneity (Santos et al., 2024). To overcome these limitations, 3D culture systems, including hydrogel, spheroid, organoid, and microfluidic chip models, have been developed. These 3D systems have enabled researchers to investigate TAM recruitment, infiltration, and polarization, as well as their effects on tumor progression, more accurately while also revealing the challenges associated with reproducing the mechanical complexity of the tumor microenvironment.

### 4.1 Hydrogel models for studying TAM–matrix interactions

Among 3D culture models, hydrogels can provide a physiologically relevant tumor microenvironment by supporting cell–cell and cell–matrix interactions, cellular migration, and cell growth (Vernerey et al., 2021; Abuwatfa et al., 2024; Suo et al., 2019). In particular, hydrogels are suitable for investigating TAM mechanobiology because they can be engineered to control properties such as stiffness, viscoelasticity, porosity, and adhesion ligand density (Charrier et al., 2018; Asadikoraye et al., 2024; Hogrebe et al., 2018). These properties can regulate macrophage infiltration and polarization and thereby influence tumor progression (Figure 3). When patient-derived primary PDAC cells and macrophages were cocultured in a PA hydrogel, the PDAC cells formed ductal colony structures resembling native pancreatic ductal tissue (Osuna de la Peña et al., 2021). In this PA-ECM hydrogel, increased fibrillar density was associated with EMT-related changes, suggesting that hydrogel models can regulate cellular phenotypes through mechanotransduction (Osuna de la Peña et al., 2021). Moreover, when macrophages were treated with CSF-1 to induce polarization, their gene expression patterns were similar to those observed in actual tumors (Osuna de la Peña et al., 2021). In a collagen-based quasi-3D model, cancer cells dynamically remodeled collagen fibers, and the resulting matrix-transmitted tensile forces directed macrophages toward cancer cells through activation of mechanosensitive ion channels (Yang et al., 2023). These findings suggest that hydrogel systems can provide a microenvironment that more accurately regulates macrophage behavior.

**FIGURE 3:**
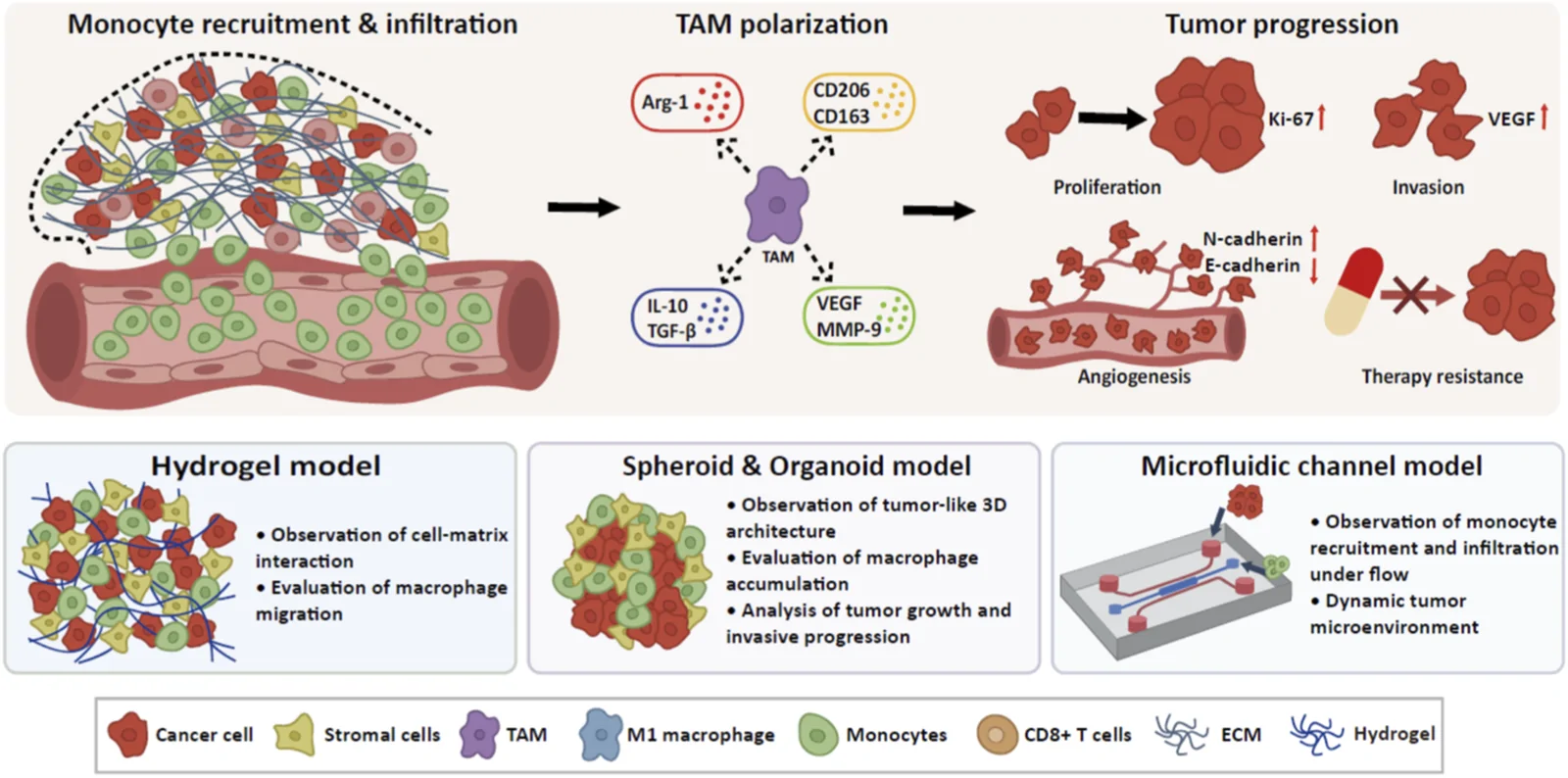
TAMs in 3D culture systems. Hydrogel-based models, spheroid and organoid models, and microfluidic chips can recapitulate cellular interactions within the tumor microenvironment. These models enable the investigation of monocyte/macrophage recruitment, TAM polarization, and TAM-mediated tumor progression, including cancer cell invasion, angiogenesis, immunosuppression, and therapeutic resistance.

Hydrogel models can also recapitulate multicellular interactions that regulate macrophage infiltration and polarization. For example, when triple-negative breast cancer patient-derived cells were encapsulated in four different hydrogels (HA-MMPx, HA + LAM, HA + COLL, and Matrigel), macrophages constituted the majority of immune cells infiltrating the tumor cell populations (Baker et al., 2022). Similarly, when melanoma patient-derived organoids were cultured in collagen gel and Matrigel, the number of PD-L1-expressing macrophages increased, indicating the establishment of an immunosuppressive tumor microenvironment (Ou et al., 2023). These findings suggest that hydrogel-based models provide useful platforms for investigating immune cell infiltration, which is difficult to recapitulate in conventional 2D culture systems.

Hydrogel models can also reproduce multicellular interactions that promote macrophage polarization. When colon cancer cells, monocytes, and CAFs were cocultured in a collagen gel, CAF-derived factors, including M-CSF, CCL2, and IL-6, attracted monocytes and promoted their differentiation into a CD163^high^ macrophage phenotype (Stadler et al., 2021). Similarly, in Matrigel containing gastric cancer organoids and splenocytes, the frequency of CD8^+^ T cell was reduced, while that of CD206^+^ F4/80^+^ macrophages increased, suggesting that an immunosuppressive environment was established (Yu X. et al., 2026). Additionally, a model consisting of ovarian cancer cells, stromal cells, and THP-1 cells was constructed using bioprinting technology, and IL-7R expressing tumor regions were enriched with CD68^+^CD206^+^ macrophages (Zheng et al., 2025). These findings indicate that hydrogel systems can recapitulate multicellular tumor–stromal–immune interactions that promote TAM-like polarization.

Changes in macrophage morphology and functional state associated with tumor progression can also be observed in hydrogel platforms. In a model recapitulating the 3D tumor niche using genipin-crosslinked collagen scaffolds, macrophages exhibited a thin, elongated morphology, suggesting M2-like polarization (Isali et al., 2021). Furthermore, the expression of IL-10, TGF-β1, Arg-1, and CD206 was substantially higher than that observed in 2D cultures (Wu et al., 2024). In addition, when hepatocellular carcinoma cells (HCCs) and macrophages were indirectly cocultured in an ECM hydrogel-based 3D tumor model, macrophages acquired a pro-inflammatory and pro-angiogenic phenotype, as indicated by upregulation of CXCL16, VEGFR2, IL8, MCP1, IL-1β, TNF-α, and MMP9 (Lim et al. 2022). Thus, 3D hydrogel systems can provide a microenvironment that promotes macrophage differentiation into distinct functional states, including M2-like phenotypes.

Macrophage polarization toward immunosuppressive TAMs is also associated with tumor-promoting effects, including increased tumor growth and proliferation and enhanced ECM remodeling, and these features can be observed in 3D hydrogel models (Zhong et al., 2024; Liu P. et al., 2025). In an indirect coculture system based on GelMA hydrogel, the expression of ECM-remodeling enzymes Mmp2 and Mmp9 and glioblastoma-associated macrophage (GAM)-related markers, including fibroblast growth factor, receptor, IL-1β, Arg-1, nitric oxide synthase 2, fibroblast growth factor receptor 1, IL-10, and IL-6, was substantially higher than that observed in 3D macrophage monocultures (Heinrich et al., 2019). In another GelMA hydrogel-based coculture system, the epithelial cell density and Ki67 expression of ovarian cancer cells increased in the presence of immunosuppressively polarized macrophages, whereas macrophage depletion decreased Ki67 and pan-cytokeratin expression (Zheng et al., 2025). Moreover, glioblastoma cell-associated markers, including SPP1, CCL2, and chitinase-3-like-1 protein, which are related to macrophage recruitment and tumor promotion and are associated with poor survival in patients with glioblastoma, were increased (Heinrich et al., 2019; Jiang et al., 2026; Ren et al., 2024; Chen et al., 2021). These findings suggest that glioblastoma cells can recruit macrophages that subsequently support tumor cell survival and growth. Similarly, when breast cancer cells were cocultured with polarized TAMs, the resulting spheroids were larger than those produced by monocultures or indirect cocultures, and the expression of Ki67, cell-cycle genes, and tumor stemness markers was increased (Xu et al., 2024).

Overall, hydrogel-based models provide ECM-like microenvironments for investigating macrophage infiltration, polarization, and TAM-mediated tumor progression. Despite these advantages, hydrogel models remain limited in their ability to fully recapitulate the complexity of the tumor microenvironment, including TAM heterogeneity and spatial variation (Table 3).

**TABLE 3:** Comparison of 3D models for studying TAMs.

3D models	Key mechanical features	Main applications for TAM	Advantages	Limitations	Ref.
Hydrogel model	Matrix composition-dependent viscoelasticity Stress relaxation	Monocyte/macrophage infiltration Matrix-dependent macrophage polarization	Tunable matrix properties Cell-matrix interactions	Difficult to represent TAM subtype heterogeneity Limited simulation of dynamic tumor mechanics, spatial variation, and vascular flow	(Baker et al., 2022; Heinrich et al., 2019)
Spheroid and organoid model	Physical confinement	Spatial analysis of TAM recruitment and infiltration	Tissue-like architecture Spatial distribution	Difficult to separate architectural and signaling effects Limited control over mechanical properties	(Martins et al., 2023; Ascheid et al., 2024)
Microfluidic chip model	Controlled fluid flow Vascular perfusion Matrix remodeling	Recruitment of circulating monocytes/macrophages TAM polarization	Dynamic control of flow Real-time observation	Difficult to separate mechanical and biochemical signaling effects Challenges with long-term culture, standardization, and reproducibility	(Hezaveh et al., 2022; Nguyen et al., 2025)

### 4.2 Spheroid and organoid models for studying spatial and functional interactions of TAMs

While hydrogel models are useful for mimicking cell–matrix interactions (Cruz-Acuña et al., 2023), organoid and spheroid models can more closely approximate the *in vivo* architecture of the tissue of origin and provide spatial interactions that are not readily captured by 2D cultures. These models can therefore better recapitulate cell proliferation, self-organization, and differentiation within the tumor microenvironment (Brancato et al., 2020; Figure 3).

TAMs, which contribute to tumor progression, accumulate near or within tumor tissues and are closely associated with poor clinical outcomes (Taufalele et al., 2023). When tumor spheroids were cocultured with macrophages, macrophages accumulated around the spheroids, similar to their distribution in tumors. In a triple-coculture model, most macrophages were located near colorectal tumor spheroids, whereas some infiltrated the spheroids (Bauleth-Ramos et al., 2020). Similarly, in a glioblastoma (GBM) multicellular tumor model, CD68^+^ macrophages accumulated not only at the spheroid surface but also within the spheroid, while fibronectin levels in the ECM were reduced, suggesting that proteolytic ECM remodeling facilitated macrophage infiltration (Martins et al., 2023). In a 3D vascularized tumor spheroid (VTS) model, CD11^+^ macrophages tended to infiltrate the VTS, and some were localized near pseudovascular regions (Ascheid et al., 2024). Moreover, in advanced 3D spheroid-based models, monocytes recirculating through blood vessels were recruited into the tumor microenvironment and exhibited stronger monocyte/macrophage signals than those observed in noncancerous models (Zhang et al., 2025). The level of GM-CSF, which is involved in monocyte recruitment, was significantly increased, and the levels of inflammatory cytokines IL-6 and IL-8 were also higher than those in the noncancerous model (Zhang et al., 2025). Additionally, in a phosphatase and tensin homolog KO gastric cancer organoid-based transplantation model, M2 macrophage and neutrophil infiltration increased, whereas CD8^+^ T-cell infiltration decreased (He et al., 2026). Macrophage infiltration can also be regulated by tumor-derived signals. In a breast cancer spheroid–monocyte coculture model, tumor-derived miR-375 was transferred to monocytes, reducing PXN and TNS3 expression and promoting macrophage infiltration (Frank et al., 2019). Collectively, these findings demonstrate that spheroid models enable the observation not only of macrophage accumulation but also of their spatial distribution and infiltration within tumor regions.

Generally, M1 macrophages exhibit pro-inflammatory and antitumor characteristics (Fang et al., 2024; Yu et al., 2024), whereas M2 macrophages exhibit anti-inflammatory and pro-tumor characteristics that can promote tumor progression (Li et al., 2025d; Kang et al., 2022). Thus, when infiltrating monocytes or macrophages acquire an M2-like phenotype, they can adopt tumor-promoting characteristics associated with TAMs. When THP-1 monocytes infiltrated MDA spheroids, CD206 and PD-L1 expression increased (Dhandapani et al., 2023). In a highly metastatic hybrid spheroid model, CD206 and TGF-β1 expression increased as the spheroids grew larger, suggesting the development of an immunosuppressive and pro-metastatic microenvironment (Seo et al., 2024). Additionally, the number of TREM2^+^ macrophages, another TAM subtype with immunosuppressive properties, increased in an Apoa1-overexpressing organoid-derived mouse tumor model, whereas the number of CD8^+^ T cells decreased, indicating the establishment of an immunosuppressive environment (Zhao et al., 2026; Molgora et al., 2020). Various signals within the tumor microenvironment can induce M2-like phenotypes in macrophages. Treatment of naive BMDMs with medium conditioned by 4T1 cells increased the expression of M2-associated marker genes, including CD206, Agr1, and Ym-1 (Wang et al., 2022). Similarly, treatment of M0 macrophages with breast cancer cell-conditioned medium increased the expression of TAM-associated markers, including CD163, TGF-β1, IL-10, and CD274, in response to tumor-derived arginine (Zhu et al., 2025).

Macrophage polarization and tumor-promoting effects arising from tumor–stroma–immune interactions can also be observed in spheroid and organoid models (Yang R. et al., 2024; Mak et al., 2025). When stromal cells were incorporated into osteosarcoma spheroids without additional stimulation, the immune population shifted toward an M2-like phenotype, with CD163^+^ macrophages representing 62.7% of the population, approximately 6-fold higher than the proportion of CD86^+^ macrophages, an M1-associated marker (Cos et al., 2024). Similarly, in another 3D model containing colorectal cancer spheroids and CAFs, CD163^+^ macrophages increased, and SPP1^+^ macrophages, which exhibit immunosuppressive properties and are positively associated with EMT markers, were strongly induced in the presence of both colorectal cancer cells and CAFs (Li et al., 2023; Qi et al., 2022; Trehan et al., 2025). These findings indicate that interactions between tumor cells and CAFs can promote macrophage differentiation toward a TAM-like phenotype. However, it remains difficult to determine whether TAM polarization and tumor progression are driven primarily by 3D architecture or by soluble factors and cell–cell signaling.

As macrophages acquire an M2-like phenotype, they can establish a tumor-supportive and pro-invasive microenvironment that promotes cancer cell proliferation, invasion, and drug resistance. In a 3D coculture model containing macrophages, breast cancer cells exhibited more prominent protrusions and a more elongated morphology, while spheroid area increased over time, indicating the development of an enhanced migratory and invasive phenotype (Gurung et al., 2025). In another model, increased collagen IV and Ki67 expression indicated that TAM-derived ECM remodeling enhanced cancer cell proliferation, while doxorubicin resistance also increased, suggesting that TAM-mediated tumor progression was successfully recapitulated (Emami et al., 2022).

Tumor expansion can also be observed in macrophage-derived tumor-supportive microenvironments in 3D coculture models. The cross-sectional areas of spheroids increased 1.9-fold and 2.6-fold in the MDA-MB-231Br and F2-7 coculture models, respectively, compared with those in the corresponding monoculture models (Song et al., 2020). Similarly, when 4T1 cancer cells were cocultured with MSC^CXCL12+/+^-primed macrophages, the number and size of 4T1 multicellular tumor spheroids increased over time, indicating that CXCL12-primed macrophages acquired TAM-like characteristics and promoted tumor progression (Babazadeh et al., 2021).

Similar findings have been observed in organoid models. The total number of duodenal adenocarcinoma (DA) organoids larger than 100 μm and Ki67 expression increased in macrophage coculture models compared with DA monoculture models (Li J. et al., 2025). In this model, tumor progression was promoted by macrophage-derived CCL5–CCR1 signaling in malignant DA cells, which suppressed apoptosis (Li J. et al., 2025). Moreover, activation of the aryl hydrocarbon receptor in macrophages by indole-3-acetic acid induced an immunosuppressive and tumor-promoting phenotype, and these macrophages promoted organoid growth when cocultured with PDAC patient-derived organoids (PDOs) (Hezaveh et al., 2022).

TAMs can also promote tumor invasiveness by inducing EMT, a process in which epithelial cells lose their strong cell–cell adhesion and stable morphology and acquire the migratory and invasive characteristics of mesenchymal cells. Decreased E-cadherin expression and increased N-cadherin and Twist1 expression were observed in tumor spheroid–macrophage coculture models (Casanova-Acebes et al., 2021; Pisano et al., 2025). Moreover, EMT-related programs were enhanced when M2 macrophages were cultured with stromal components in tumoroids, resulting in increased tumor spheroid and organoid invasiveness (Casanova-Acebes et al., 2021; Chen Y. et al., 2023; Yu K. K. H. et al., 2026; Burkhard et al., 2025). TAMs not only induce tumor EMT but can also increase tumor cell stemness and aggressiveness, which are associated with chemoresistance (Jiang et al., 2023; Wang Y. et al., 2024; Cascio et al., 2021). In an ovarian cancer spheroid model enriched in aldehyde dehydrogenase, a cancer stem cell marker, cancer cells exhibited chemoresistance to carboplatin (Raghavan et al., 2019). Similarly, tumoroids cocultured with M2 macrophages showed higher expression of cancer stem cell markers, including CD44 and CD90, greater resistance to carboplatin, and higher cell viability than tumoroids cultured alone (Burkhard et al., 2025). In addition, TAM-containing microenvironments were associated with therapy-resistant phenotypes and, in some models, increased tumor stemness (Gurung et al., 2025; Jiang et al., 2023; Emami et al., 2021).

Overall, spheroid and organoid models provide valuable platforms for studying TAM recruitment, infiltration, polarization, and tumor-promoting functions by recapitulating spatial distribution, 3D tissue-like architecture, and physical confinement. However, as with hydrogel models, these systems have limited ability to capture TAM heterogeneity and to distinguish the effects of mechanical regulation from those of soluble factors and cell–cell signaling (Table 3).

### 4.3 Microfluidic chip models for investigating dynamic TAM–tumor environment interactions

Unlike spheroid and organoid models, which primarily mimic tumor architecture and aspects of native tissue organization, microfluidic chip models provide a dynamic microenvironment that more precisely recapitulates cell migration, immune cell infiltration, and cell–cell and cell–matrix interactions within the tumor microenvironment (Maulana et al., 2024; Ravi et al., 2025; Figure 3). In particular, tumor-on-a-chip models can incorporate fluid flow and chemokine gradients, features that are difficult to reproduce in hydrogel, spheroid, and organoid models. From this perspective, microfluidic chip models represent potentially useful platforms for studying TAM regulation (Polacheck et al., 2013; Wan et al., 2020). In an immunocompetent tri-culture osteosarcoma model in which osteosarcoma cells, human umbilical vein endothelial cells, and M2 macrophages were integrated into a microfluidic system, cell viability and the M2 phenotype were maintained, and the model successfully recapitulated the gradual development of an anti-inflammatory and immunosuppressive tumor microenvironment over time (Jaiswal et al., 2026). Similarly, in another microfluidic chip model in which pancreatic cancer cells and stromal cells were cocultured with macrophages, pancreatic cancer cells grew rapidly, while macrophages and pancreatic stellate cells localized near the tumor cells, resembling the organization of the native tumor microenvironment (Haque et al., 2022). Macrophages also exhibited pro-tumorigenic and M2-like phenotypes, as indicated by CD163 expression, suggesting that the microfluidic model recapitulated not only key features of the PDAC tumor microenvironment but also characteristics of the TAM-associated microenvironment (Haque et al., 2022).

A major advantage of microfluidic platforms is their ability to model the recruitment of circulating monocytes and macrophages and their subsequent infiltration into tumor tissue. In one model, endothelial cells and fibroblasts were embedded in a fibrin hydrogel within a microfluidic chamber to generate self-assembled microvascular networks, after which a tumor spheroid containing MDA-MB-231 tumor cells, fibroblasts, and macrophages was introduced to establish a tumor-like microenvironment (Nguyen et al., 2025). When primary human monocytes were subsequently perfused through the vascular channel, their recruitment toward the tumor spheroid was stronger than that observed in spheroids without macrophages (Nguyen et al., 2025). Recirculating monocytes were recruited through CCL2/CCL7 signaling mediated by CD163-expressing macrophages, suggesting that macrophages within the spheroid contributed to the recruitment of circulating monocytes (Nguyen et al., 2025). Similarly, in a PDAC organ-on-a-chip model, a fibrotic tumor stroma (FibTS) containing PDAC cells (FibTS/PDAC^+^) attracted more migrating macrophages than either a collagen-based stroma containing PDAC cells (ColTS/PDAC^+^) or FibTS without PDAC cells (FibTS/PDAC^−^) (Karim et al., 2025). Analysis of the RNA expression profiles of macrophages infiltrating the FibTS/PDAC^+^ environment showed an increased proportion of CD163^+^ macrophages, with M2-like TAMs predominating over M1-like TAMs within the PDAC colony (Karim et al., 2025). Additionally, genes associated with NF-κB, TNF, S100, and TLR signaling pathways were upregulated, suggesting the establishment of an immunosuppressive tumor microenvironment (Karim et al., 2025). Taken together, these findings suggest that FibTS promoted macrophage recruitment and induced polarization toward an M2-like TAM phenotype, thereby contributing to the establishment of an immunosuppressive PDAC microenvironment that may promote tumor progression (Karim et al., 2025).

Microfluidic chip models can also be used to analyze TAM-induced changes in tumor cell phenotypes and behaviors. In a coculture model containing tumor cells, CAFs, and macrophages within a 3D organotypic breast tumor microenvironment-on-a-chip system, immune evasion-related pathways were upregulated, and patients with higher expression of these pathways had poorer overall survival (Ravi et al., 2025). These findings suggest that macrophages can enhance tumor cell aggressiveness and immune evasion in the presence of CAFs, thereby facilitating tumor progression (Ravi et al., 2025). Similarly, in a microfluidic spheroid coculture model, TAM-derived TGF-β1 reduced E-cadherin expression and enhanced T47D cell migration, indicating that TAMs can shift tumor cells toward a more migratory phenotype (Yuan et al., 2019).

Macrophages can also alter cancer cell migratory behavior through interactions with the ECM. For example, in a microfluidic device containing a collagen I-based ECM, macrophages increased the speed, directedness, and invasiveness of cancer cell migration (Li et al., 2017). Migration speed and directedness were enhanced by macrophage-derived TGF-β1, while TNF-α and TGF-β1 increased the expression of matrix-degrading enzymes (Li et al., 2017). Additionally, in a microfluidic premetastatic niche model containing extravasated monocytes, vascular permeability increased because monocyte-derived MMP9 disrupted the endothelial barrier (Kim et al., 2019). Preinvasion macrophages also promoted cancer cell invasiveness by remodeling the basement membrane and collagen matrix (Kim et al., 2019). Moreover, in a 3D biomimetic microfluidic GBM model, GBM cells polarized macrophages toward an M2-like phenotype, and these polarized macrophages promoted endothelial cell sprouting and capillary network formation, whereas M1 macrophages inhibited angiogenesis (Cui et al., 2018).

Taken together, these studies demonstrate that microfluidic chip models are valuable platforms for investigating TAM recruitment, extravasation, polarization, and function while enabling the analysis of TAM-mediated matrix remodeling, vascular barrier integrity, and vascular architecture. Because microfluidic chip models can simultaneously recapitulate fluid flow, shear stress, matrix stiffness, and biochemical gradients, however, it can be difficult to disentangle the effects of biochemical signaling from those of mechanical cues. In addition, technical challenges related to long-term culture, standardization, and reproducibility remain important limitations (Table 3).

## 5 TAM-targeted therapies

TAMs, particularly M2-like TAMs, promote tumor cell proliferation, angiogenesis, invasion, metastasis, and therapy resistance by establishing an immunosuppressive tumor microenvironment, thereby contributing to poor clinical outcomes. Accordingly, various therapeutic strategies targeting TAMs have been actively investigated for their potential to suppress tumor progression. These strategies can be broadly categorized into two groups: TAM depletion and TAM reprogramming.

### 5.1 Strategies for direct or selective TAM depletion

#### 5.1.1 Blocking TAM recruitment and survival

TAM depletion leads to reduction of tumor-promoting macrophage populations within tumor microenvironment by limiting their pro-tumorigenic properties (Figure 4). One strategy for regulating TAMs is to reduce their accumulation within tumors by blocking the CSF-1/CSF-1R signaling axis, which is critical for macrophage survival and differentiation (Sehgal et al., 2021). Indeed, several studies have demonstrated that inhibition of CSF-1 signaling reduces the number of intratumoral macrophages, particularly M2-like TAMs, while remodeling the tumor microenvironment toward a less immunosuppressive state. Emactuzumab (RG7155), a CSF-1R–blocking antibody, selectively depleted CSF-1–differentiated macrophages by inducing the death of CD163^+^ macrophages and suppressed CD4^+^ and CD8^+^ T-cell proliferation (Ries et al., 2014). Furthermore, the numbers of CSF-1R^+^ cells and CD68^+^CD163^+^ macrophages were reduced in a variety of solid tumors treated with emactuzumab, indicating that macrophage depletion also occurred *in vivo* (Ries et al., 2014). Following chemotherapy, residual tumor cells can produce TGF-β, which upregulates PD-L1 and promotes tumor cell-derived CSF-1–mediated TAM recruitment, thereby creating an immunosuppressive tumor microenvironment that ultimately contributes to therapy resistance and recurrence (Chen et al., 2022). Pexidartinib, another CSF-1R inhibitor, blocked the TGF-β/PD-L1/CSF-1 signaling axis and reduced TAM recruitment. Cotreatment with pexidartinib and the TGF-β signaling inhibitor and therapeutic agent oxaliplatin (OXP) reduced tumor growth and CD163^+^ macrophage populations more effectively than pexidartinib treatment alone (Chen et al., 2022). This combination also increased PD-L1 expression and CD8^+^ T-cell numbers while reducing OXP-induced Smad phosphorylation and the abundance of immunosuppressive regulatory T cells, suggesting that the tumor microenvironment shifted toward a more antitumorigenic state (Chen et al., 2022). Similar effects were observed in a 3D collagen model, in which BLZ945 treatment downregulated CCR2 and CSF-1 signaling, leading to a reduction in the CD163^+^ macrophage population (Stadler et al., 2021). Notably, macrophage recruitment was also regulated by stiffness-dependent CSF-1 signaling. CSF-1 expression was higher in stiff gels than in soft gels, resulting in greater macrophage recruitment, whereas inhibition of CSF-1R on macrophages significantly attenuated this stiffness-associated recruitment (Taufalele et al., 2023).

**FIGURE 4:**
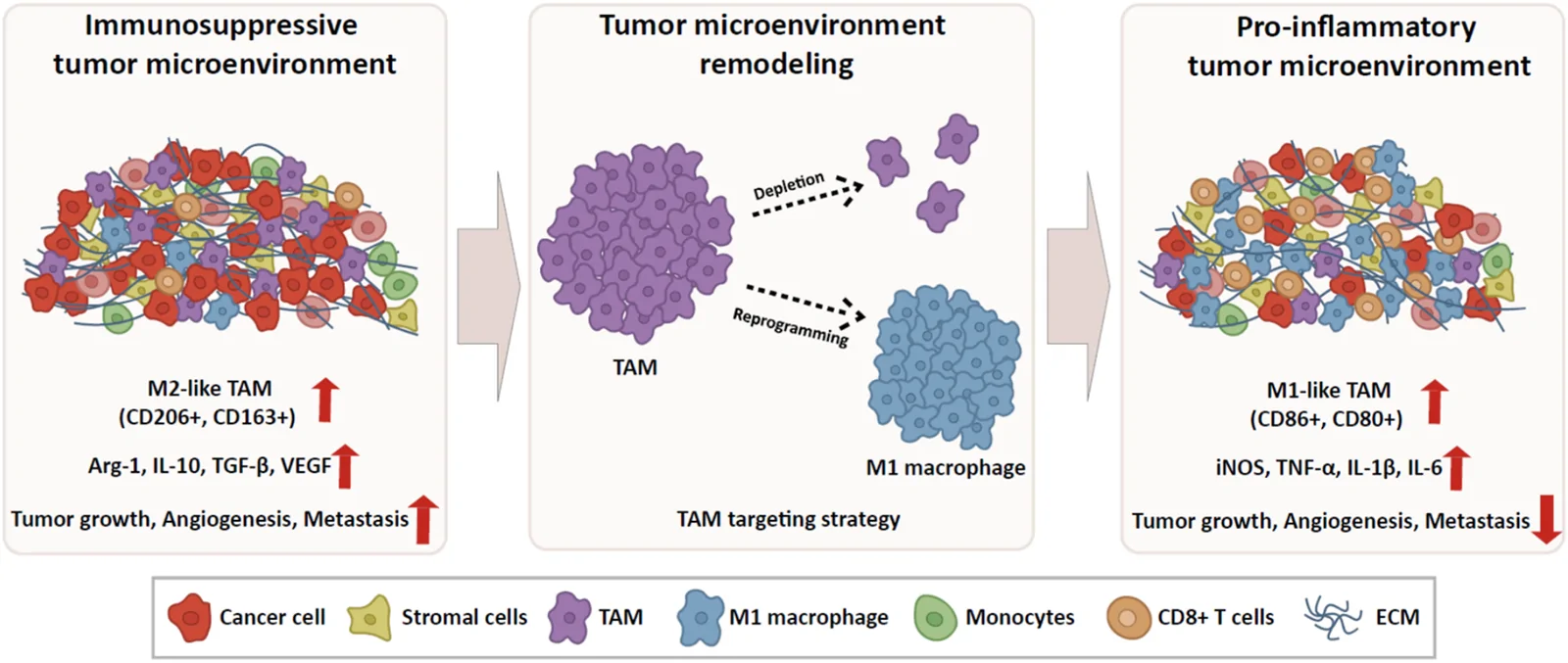
TAM-targeted therapies. Therapeutic strategies targeting TAMs include direct depletion of tumor-promoting macrophages and reprogramming of TAMs toward antitumor phenotypes. These approaches aim to overcome TAM-mediated immunosuppression and remodel the tumor microenvironment toward a more pro-inflammatory state, thereby limiting tumor progression and enhancing the efficacy of cancer therapies.

Whereas CSF-1 inhibitors primarily target TAM survival and maintenance, blockade of the CCL2/CCR2 axis can inhibit the recruitment of circulating monocytes into the tumor microenvironment. In a CCR2 KO model, tumor growth was reduced by 60%, CD11b^+^Ly6C^high^ inflammatory monocyte infiltration was almost completely blocked, and the expression of inflammatory chemokines, including CCL3 and CCL5, was suppressed (Yang et al., 2020). Similarly, blocking stromal cell-derived CCL2 signaling reduced monocyte migration and tumor mass (Ren et al., 2012). Beyond suppressing monocyte recruitment, CCL2 deficiency also reduced M2-like phenotypes, including CD206, VEGF, CD163, and IL-4/IL-13-associated phenotypes (Tu et al., 2021). Based on these mechanistic findings, pharmacological blockade of the CCL2–CCR2 axis has been investigated as a strategy to suppress monocyte/macrophage recruitment and promote antitumor effects. A small-molecule CCR2 inhibitor (CCR2i) attenuated the immunosuppressive tumor microenvironment by reducing TAM infiltration and regulatory T-cell numbers in MC38 metastatic colorectal cancer (mCRC) liver tumors while increasing tumor-infiltrating lymphocytes and CD8^+^ T-cell activation (Grossman et al., 2018). Cotreatment with CCR2i and FOLFOX significantly reduced tumor progression, liver tumor weight, and tumor burden while increasing survival (Grossman et al., 2018). Additionally, in a CCL2-secreting metastatic breast cancer model, treatment with an anti-CCL2 antibody reduced lung metastases, circulating tumor cells, cancer cell motility, and the total intratumoral number of CD11b^+^CD11c^+^CD206^+^MHCII^+^ cells, indicating that cancer cell intravasation into blood vessels was limited (Bonapace et al., 2014). In contrast, discontinuation of CCL2 blockade increased the number of infiltrating monocytes and macrophages in lung metastases and induced local vascular growth and metastatic proliferation (Bonapace et al., 2014). Taken together, these findings indicate that targeting the CCL2/CCR2 axis can reduce TAM accumulation and alleviate the pro-tumorigenic, immunosuppressive tumor microenvironment by blocking monocyte and macrophage recruitment. However, macrophage migration was restored when interstitial flow was applied in a microfluidic chip model (Lee S. W. L. et al., 2020). This finding indicates that although inhibition of CCL2/CCR2 signaling can reduce macrophage infiltration and accumulation, its effects may be modified by mechanical cues such as interstitial flow (Table 4).

**TABLE 4:** Comparison of TAM depletion and TAM reprogramming strategies.

Category	TAM depletion			TAM reprogramming			
Therapeutic strategy	CSF-1/CSF-1R signaling blockade	CCL2/CCR2 signaling blockade	Selective TAM subtype targeting	Extracellular receptor and tumor microenvironment signaling modulation	Intracellular signaling modulation	Metabolic modulation	NP- and carrier-mediated delivery
Therapeutic agent or approach	Emactuzumab Pexidartinib BLZ945	CCR2 inhibitor Anti-CCL2 antibody	Trabectidin Clodronate liposomes mFRβ CAR-T cells SQLE targeting	Bosentan E−301 TIM3/VISTA blockade Cilengitide	Wnt/β-catenin signaling inhibition exoASO-STAT CD206 receptor	IRG1/WDR4 targeting D-lactate NPs Succinate-loadedmicroparticles	Pirfenidone-loaded micelles DMS/DOX-loaded NPs
Effects on TAM	Inhibition of macrophage recruitment, infiltration, accumulation, and overall abundance			Conversion of immunosuppressive and pro-tumoral TAMs into pro-inflammatory and antitumoral TAMs			
Limitations	Limited characterization of mechanical properties Incomplete evaluation in mechanically relevant 3D models			Limited mechanobiological characterization of TAM reprogramming Unclear causal link between matrix normalization and TAM phenotypic conversion			

#### 5.1.2 Direct depletion and selective targeting of TAMs

While inhibition of the CSF-1/CSF-1R and CCL2/CCR2 signaling pathways indirectly reduces TAM abundance by blocking monocyte/macrophage recruitment and promoting the loss of TAMs, direct depletion may provide a more effective strategy for eliminating tumor-promoting macrophages. Treatment with trabectedin (ET-743), a DNA-binding chemotherapeutic agent that inhibits cell cycle progression, reduced the proportion of CD11b^+^F4/80^+^ macrophages from 60% to 13% (Germano et al., 2013). Notably, immature macrophages (F4/80^+^ Ly6C^int/high^) accounted for 75% of the remaining TAM population, suggesting that trabectedin interfered with the maturation of tumor-recruited monocytes into macrophages (Germano et al., 2013). This depletion was attributed to the selective induction of apoptosis in monocytes and macrophages by trabectedin through activation of the TRAIL receptor-mediated extrinsic apoptotic pathway (Germano et al., 2013). Another direct depletion strategy involves clodronate liposomes (CLs), which act as macrophage-depleting agents by inducing apoptosis following phagocytosis (Lu et al., 2023; Choi et al., 2024). Mammary gland tissue-resident macrophages (MGTRMs) are a major source of TAMs during early tumor development and contribute to the proliferation and angiogenesis of triple-negative breast cancer (Hirano et al., 2023). Treatment with CLs effectively reduced MGTRM abundance and tumor growth, while combined treatment with doxorubicin and CLs markedly reduced triple-negative breast cancer recurrence and lung and liver metastases without causing obvious adverse effects, including weight loss (Hirano et al., 2023).

In addition to broad depletion of the total macrophage population, strategies have been developed to selectively target specific TAM subtypes. Diphtheria toxin (DT), which selectively eliminates DTR-expressing cells by inhibiting protein synthesis, has been used to deplete CD169-expressing tumor-resident macrophages (Casanova-Acebes et al., 2021; Robinson et al., 2023). When CD169-DTR mice were treated with DT, CTLA-4 and CD73 expression in regulatory T cells decreased, whereas the total numbers of CD3^+^ and IFNγ^+^TNF^+^CD8^+^ T cells increased, suggesting attenuation of the immunosuppressive tumor microenvironment (Casanova-Acebes et al., 2021). Another TAM subtype, FRβ^+^, which exhibit an M2-like phenotype associated with suppression of antigen-specific T-cell function, was targeted using mFRβ CAR-T cells in ID8 tumor-bearing mice (Rodriguez-Garcia et al., 2021). Depletion of this TAM population significantly delayed tumor growth and prolonged survival. In addition, IL-10 expression in myeloid cells, including TAMs, decreased, whereas TNF-α expression increased, indicating that the immunosuppressive tumor microenvironment was shifted toward a more pro-inflammatory state (Rodriguez-Garcia et al., 2021). Similarly, targeting squalene epoxidase (SQLE), which is strongly associated with survival in HCC patients, led to depletion of TREM2+ TAMs (Chu et al., 2025). SQLE expression was positively correlated with the relative abundance of TREM2^+^ TAMs among TAM subtypes. In HCC models with SQLE knockdown, SPP1^+^TREM2^+^ TAM infiltration and tumor volume were reduced, whereas IFN-γ+CD8^+^ T-cell activation increased (Chu et al., 2025). Because SPP1^+^ macrophages and FAP^+^ fibroblasts contribute to ECM remodeling and promote the formation of desmoplastic regions within the tumor microenvironment and resistance to immunotherapy, targeting SPP1^+^ macrophages may represent a potential strategy for attenuating ECM remodeling (Qi et al., 2022).

### 5.2 TAM reprogramming

#### 5.2.1 Extracellular receptor- and tumor microenvironment signaling-based reprogramming

Another TAM-targeted therapeutic strategy is to reprogram immunosuppressive and tumor-promoting TAMs into pro-inflammatory and antitumor TAMs (Figure 4). TAM phenotypes and functions can be regulated by extracellular signals from cancer cells, stromal cells, and other immune cells within the tumor microenvironment, which can promote immunosuppressive and tumor-supportive states. Therefore, blocking these signals may reprogram pro-tumoral TAMs toward antitumoral phenotypes. For example, EPO/EPOR signaling has been shown to regulate TAM phenotypes. Erythropoietin (EPO), a cytokine that regulates red blood cell production, can promote an immunosuppressive tumor microenvironment and is highly expressed in immunoregulatory macrophages within the HCC tumor microenvironment (Chiu et al., 2025). In an EPOR KO model, activated CD8^+^ T cells, including TNF-α–producing CD8^+^ effector memory T cells, and MHCII^+^CD86^+^ macrophages were increased compared with those in control mice. Moreover, combined EPOR deficiency and anti-PD-1 treatment resulted in complete tumor regression (Chiu et al., 2025). In another receptor-targeting approach, E−301, a trastuzumab-sialidase fusion protein, was administered to a HER2-positive tumor model enriched with TAMs that strongly expressed sialic acid-binding immunoglobulin-like lectin-E (Siglec-E), an immunosuppressive receptor (Stanczak et al., 2022). E−301 treatment increased the expression of antitumor-related genes, including *IL-1β*, *TNF*, *CD80*, and *Arg2*, as well as the abundance of MHCII^+^CD206^−^ macrophages, resulting in an increased M1/M2 ratio (Stanczak et al., 2022). This reprogramming effect was mediated by E−301-induced desialylation through the sialoglycan–Siglec axis within the tumor microenvironment, thereby delaying tumor growth and enhancing antitumor immunity (Stanczak et al., 2022).

Beyond targeting cytokine–receptor signaling, TAM reprogramming can also be achieved by combining immune checkpoint blockade with chemotherapy. Treatment with the immunogenic cell death (ICD)-inducing chemotherapeutic agent paclitaxel together with TIM3 or VISTA antibodies reduced the number of TIM3^+^VISTA^+^CSF1R^+^ TAMs and increased the population of pro-inflammatory M1-like (MHCII^HIGH^CSF1R^LOW^) TAMs to levels exceeding those of M2-like TAMs in an LCC tumor model (Vanmeerbeek et al., 2024). TIM3 and VISTA are immune checkpoint proteins that suppress T-cell activation and contribute to the establishment of an immunosuppressive tumor microenvironment (Dixon et al., 2021; Lin et al., 2024). Similarly, in a TIM3^+^VISTA^+^ TAM-rich melanoma model, treatment reduced both TAM abundance and tumor volume without altering CD8^+^ T-cell numbers, indicating that the reduction in tumor size was primarily associated with reprogramming TAMs from an anti-inflammatory toward a pro-inflammatory state rather than with increased T-cell infiltration (Vanmeerbeek et al., 2024).

Cell–matrix adhesion receptors can also mediate TAM reprogramming. Integrin α_v_β_3_, which is highly expressed in angiogenic endothelial cells, regulates α_v_β_3_ integrin–Src–PI3K–YAP signaling during endothelial cell–macrophage interactions (Cui et al., 2018). Dual inhibition with the integrin α_v_β_3_ inhibitor cilengitide and the TGF-βR1 inhibitor LY-364947 reduced the Arg-1/iNOS ratio and endothelial sprouting and capillary formation, producing an anti-angiogenic effect (Cui et al., 2018). These findings suggest that blockade of integrin α_v_β_3_-mediated signaling can suppress the pro-angiogenic functions of TAMs and GBM vascularization. Although integrin-mediated signaling represents a potential mechanism for TAM reprogramming, whether matrix properties directly regulate this process remains insufficiently investigated. Further studies are therefore needed to determine how matrix stiffness, density, and composition influence the conversion of pro-tumoral TAMs toward antitumoral phenotypes.

#### 5.2.2 Intracellular signaling-based TAM reprogramming

In addition to extracellular signals within the tumor microenvironment, intracellular signaling pathways in TAMs can also drive TAM reprogramming and therefore represent potential therapeutic targets. For example, inhibition of Wnt/β-catenin signaling in M2-like TAMs downregulated IL-10 expression and increased TNF expression (Sarode et al., 2020). Similarly, treatment with XAV939, a small molecule that inhibits Wnt/β-catenin signaling, suppressed primary tumor growth and metastasis in lung tumor models by reprogramming pro-tumorigenic TAMs toward an antitumorigenic phenotype (Sarode et al., 2020). Targeting STAT6, a key transcription factor involved in M2-like and immunosuppressive macrophage polarization, also induced TAM reprogramming. In M2-differentiated monocyte-derived macrophages (MDMs), treatment with exoASO-STAT6, an exosome-delivered antisense oligonucleotide targeting STAT6, downregulated M2-associated genes, including CD163, CD206, and TGF-β1, while increasing the expression of M1-associated genes, including IL-12β, NOS2, and IL-1β (Kamerkar et al., 2022). Consistent with these changes, tumor volume and growth were reduced and survival was improved in the tumor model, accompanied by increased CD8^+^ T-cell infiltration and reduced regulatory T-cell (Treg) populations, suggesting that STAT6 targeting remodeled the tumor microenvironment by enhancing antitumor immune responses (Kamerkar et al., 2022). Because high STAT6 expression in patients with HCC was associated with poor survival, these findings suggest that targeting STAT6 signaling in macrophages may represent a potential therapeutic strategy (Kamerkar et al., 2022). Targeting CD206, a receptor associated with M2-like macrophages, can also modulate the M2-like phenotype. The CD206-targeting peptide RP-182 reduced CD206 expression while increasing the number of CD86^+^ cells, and the expression of IL-1β, IL-12β, and TNF-α was increased in the overall macrophage population, particularly in CD86^+^ cells (Jaynes et al., 2020). Moreover, reductions in the immunosuppressive markers PD-1 and SIRP-α indicated that TAMs were reprogrammed toward a more pro-inflammatory phenotype (Jaynes et al., 2020). This effect was mediated by specific binding of RP-182 to CD206, which activated NF-κB-dependent TNF-α signaling through the GRB2–RAC1/CDC42–PAK1 signaling axis (Jaynes et al., 2020).

Together, these studies indicate that immunosuppressive TAMs can be reprogrammed toward pro-inflammatory phenotypes by targeting intracellular signaling pathways and macrophage-associated receptors. However, it remains unclear whether mechanical cues can induce TAM reprogramming by regulating intracellular signaling pathways in TAMs.

#### 5.2.3 Metabolic reprogramming of TAMs

TAMs can also be reprogrammed by modulating their metabolic state. Immune-responsive gene 1 (Irg-1) is an inflammation-inducible metabolic enzyme that produces itaconate, a metabolite associated with the immunoregulatory functions of macrophages (Cyr et al., 2024). In tumor-bearing Irg1^−/−^ mice, the populations of tumor-infiltrating monocytes and CD8^+^ T cells were increased, and M1-like TAMs (iNOS^+^F4/80^+^CD11b^+^CD45^+^) were more abundant (Chen Y.-J. et al., 2023). Additionally, the expression of the pro-inflammatory genes TNF-α, IL-6, and IL-1β was increased, whereas that of the anti-inflammatory genes Arg1, CD206, and IL-10 was reduced (Chen Y.-J. et al., 2023). In a pancreatic tumor model resistant to T cell-based immunotherapy, coculture with Irg1^−/−^ macrophages significantly increased the populations of M1-like TAMs and CD8^+^ T cells, while reducing tumor cell proliferation and tumor weight (Chen Y.-J. et al., 2023). Similarly, *WDR4*, which is highly expressed in HCC-associated TAMs and is associated with poor prognosis, has been implicated in TAM metabolic reprogramming. Coculture of WDR4-KO THP-1 cells or BMDMs with HCC cells suppressed tumor cell proliferation and reduced tumor size and weight (Liu J. et al., 2025). In addition, the antitumoral phenotype markers Nos2, IL-1β, IL-12α, CSF3R, CXCR2, TNF, FPR1, and CD80 were highly expressed, and the number of antitumoral macrophages increased, whereas that of pro-tumoral macrophages decreased (Liu J. et al., 2025). This effect was mediated by ATP-binding cassette transporter A1 (ABCA1)-dependent cholesterol efflux, which altered cholesterol homeostasis in macrophages and promoted a shift in the TAM phenotype (Liu J. et al., 2025).

In addition to targeting the endogenous metabolic state of TAMs, direct delivery of exogenous metabolites can also reprogram M2-like TAMs. Based on previous findings that D-lactate enhances pathogen clearance by Kupffer cells, a study investigated whether D-lactate could promote phagocytosis, a function associated with M1-like macrophages, and reprogram M2 TAMs toward an M1-like phenotype (McDonald et al., 2020; Han et al., 2023). When D-lactate-containing NPs were injected into HCC-bearing mice, F4/80^+^Arg1^+^ and F4/80^+^CD206^+^ M2 TAMs were reduced, whereas F4/80^+^iNOS^+^ and F4/80^+^CD86^+^ M1 TAMs were increased (Han et al., 2023). Additionally, the numbers of immunosuppressive cells, including myeloid-derived suppressor cells (MDSCs) and Tregs, were decreased, along with the expression of the immunoinhibitory factors IL-10, TGF-β, and IL-4, whereas the numbers of immunostimulatory cells increased. These changes ultimately enhanced tumor apoptosis and prolonged mouse survival (Han et al., 2023).

Similarly, treatment with succinate-loaded tumor cell-derived microparticles (SMPs) produced antitumoral effects. Because tumor cell-derived succinate is known to promote M2 macrophage polarization, whereas intracellular succinate is associated with M1-like macrophage metabolism, researchers hypothesized that SMPs could reprogram M2 TAMs (Wu et al., 2020; Lu et al., 2025). Injection of SMPs into tumor-bearing mice decreased M2 markers and increased M1 markers, thereby reducing tumor size and prolonging survival in a macrophage-dependent manner (Lu et al., 2025). In addition, the metabolic profile of BMDMs shifted toward an M1-like phenotype, characterized by increased glycolytic metabolism and decreased mitochondrial oxidative metabolism, indicating that succinate was directly delivered to macrophages and induced an M1-like phenotype through metabolic reprogramming (Lu et al., 2025).

These findings demonstrate that metabolic regulation of TAMs represents a potential therapeutic strategy for inducing TAM reprogramming. However, further studies are needed to clarify whether interactions between the mechanical properties of the tumor microenvironment and TAM metabolism can induce TAM reprogramming and promote antitumoral functions.

#### 5.2.4 Nanoparticle-based reprogramming of TAMs

Because the efficiency of therapeutic delivery is limited by the dense and heterogeneous tumor microenvironment, nanoparticle (NP)-based delivery systems have been developed to selectively deliver therapeutic agents and remodel the immunosuppressive microenvironment toward a pro-inflammatory state. For example, loading dexamethasone (DMS) into NPs improved their distribution and delivery efficiency. In an aggressive 4T1 tumor model, NPs loaded with DMS and aconityl-doxorubicin, a doxorubicin prodrug, reduced the population of M2 TAMs (F4/80^+^CD206^+^) and the expression of immunosuppressive factors while increasing the population of M1 TAMs (F4/80^+^CD80^+^) and the expression of pro-inflammatory cytokines (Chen et al., 2025). This effect was attributed to DMS-mediated enhancement of doxorubicin nuclear entry, which increased its intranuclear accumulation (Chen et al., 2025). Beyond conventional chemotherapy, which can disrupt tumor cell proliferation and survival, similar effects have been observed with pirfenidone, a fibrosis inhibitor. Treatment of 4T1 tumors with pirfenidone-loaded polymeric micelles in combination with an immune checkpoint inhibitor decreased the M2 phenotype and increased the M1 phenotype without altering the overall macrophage population, indicating that pirfenidone induced a phenotypic shift from M2-like toward M1-like macrophages (Mpekris et al., 2023). Furthermore, tumor stiffness was reduced and tumor perfusion was improved. These findings suggest that TAM reprogramming may contribute to ECM remodeling and tumor mechanical normalization. However, the direct contribution of reprogrammed TAMs to changes in tumor mechanical properties remains to be established (Table 4).

## 6 Conclusion and future perspectives

This review summarizes the diverse roles of TAMs in tumor progression and examines how their recruitment, infiltration, polarization, and functions are regulated by the mechanical properties of the tumor microenvironment. TAMs originate either from circulating monocytes recruited into the tumor microenvironment or from tissue-resident macrophages already present within the tissue. TAM recruitment, infiltration, and polarization toward an M2-like phenotype can be induced by tumor-derived signals or mechanical cues, including ECM stiffness and matrix remodeling. Despite increasing understanding of monocyte-derived and tissue-resident TAMs, several questions remain unresolved. It is still unclear how their functional programs vary across tumor types and stages, how these populations cooperate to regulate stromal remodeling and immune responses, and how mechanical properties of the tumor microenvironment, including ECM stiffness and vascular remodeling, influence their differentiation, plasticity, and therapeutic responsiveness.

During tumor progression, the ECM becomes stiffer due to increased accumulation of collagen and fibronectin, thereby promoting the recruitment and infiltration of monocytes and macrophages into the tumor microenvironment and driving their polarization toward M2-like TAM phenotypes. These TAM behaviors can be reproduced in 3D culture systems, including hydrogel, organoid and spheroid, and microfluidic chip models, which are suitable for mimicking complex interactions among cellular components of the tumor microenvironment, as well as relevant mechanical properties such as matrix composition, spatial organization, and interstitial flow. Although recent studies have investigated how mechanical cues regulate TAM behavior, the relative contributions of mechanical and biochemical signals to TAM localization, phenotypic changes, and ECM remodeling remain to be determined.

Future research should also address how TAM heterogeneity can be better represented in 3D culture systems, as this remains a common limitation across all three model types. Furthermore, TAM subtype-specific targeting strategies should be evaluated in mechanically tunable 3D culture systems to improve therapeutic precision and efficacy. Investigating the relationships between mechanosensitive signaling networks, such as integrin–FAK–YAP/TAZ and RhoA–ROCK–actomyosin signaling, and TAM behavior using 3D models may provide a better understanding of how mechanical cues regulate TAM biology. Such approaches will help clarify the complex regulation of TAM biology and facilitate the development of therapeutic strategies that combine TAM targeting with modulation of the mechanical tumor microenvironment.
